# Antimicrobial Metal Nanomaterials: From Passive to Stimuli‐Activated Applications

**DOI:** 10.1002/advs.201902913

**Published:** 2020-04-06

**Authors:** Samuel Cheeseman, Andrew J. Christofferson, Rashad Kariuki, Daniel Cozzolino, Torben Daeneke, Russell J. Crawford, Vi Khanh Truong, James Chapman, Aaron Elbourne

**Affiliations:** ^1^ School of Science College of Science Engineering and Health RMIT University Melbourne VIC 3001 Australia; ^2^ Nanobiotechnology Laboratory School of Science College of Science Engineering and Health RMIT University Melbourne VIC 3001 Australia; ^3^ School of Engineering RMIT University Melbourne VIC 3001 Australia; ^4^ Food Science and Technology Bundoora Campus School of Science College of Science Engineering and Health RMIT University Melbourne VIC 3086 Australia

**Keywords:** antimicrobial metal nanomaterials, bacteria, fungus, nanoparticles

## Abstract

The development of antimicrobial drug resistance among pathogenic bacteria and fungi is one of the most significant health issues of the 21st century. Recently, advances in nanotechnology have led to the development of nanomaterials, particularly metals that exhibit antimicrobial properties. These metal nanomaterials have emerged as promising alternatives to traditional antimicrobial therapies. In this review, a broad overview of metal nanomaterials, their synthesis, properties, and interactions with pathogenic micro‐organisms is first provided. Secondly, the range of nanomaterials that demonstrate passive antimicrobial properties are outlined and in‐depth analysis and comparison of stimuli‐responsive antimicrobial nanomaterials are provided, which represent the next generation of microbiocidal nanomaterials. The stimulus applied to activate such nanomaterials includes light (including photocatalytic and photothermal) and magnetic fields, which can induce magnetic hyperthermia and kinetically driven magnetic activation. Broadly, this review aims to summarize the currently available research and provide future scope for the development of metal nanomaterial‐based antimicrobial technologies, particularly those that can be activated through externally applied stimuli.

## Graphical abstract

Antimicrobial resistance remains a significant health issue. Recently, metal nanomaterials have emerged as promising antimicrobials. This review provides an overview of metal nanomaterials, detailing their synthesis, properties and antimicrobial activity. The antimicrobial mechanisms of these nanomaterials are discussed in the context of passive and stimuli‐responsive activity. The review summarizes this area of research and provides direction for future studies.

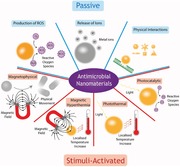

## 1 Introduction

Antimicrobial resistance (AMR) is one of the most significant health‐related issues of the 21st century.^[^ ^1^, ^2^, ^3^ ^]^ Despite continued, thorough investigations into the development of new chemical classes of antibiotics, scientific progress has been unable to contend with the rapid rise of bacterial resistance.^[^ ^1^, ^3^, ^4^ ^]^ For bacteria, several mechanisms of resistance have evolved, including decreased membrane permeability,^[^ ^5^ ^]^ overexpression of specific efflux pumps,^[^ ^6^ ^]^ the development of mechanisms to degrade or alter the conventional antibiotic,^[^ ^7^ ^]^ and the biological differentiation of the antibiotic target site.^[^ ^8^ ^]^ Bacteria possessing just one of these resistance mechanisms can be treated through an alternative class of antibiotic, however, it is becoming increasingly common for single strains of bacteria to simultaneously possess the genes for more than one of these resistance mechanisms; these bacteria are often termed “superbugs.” The rapid development of bacterial antibiotic resistance has been expedited by the life‐cycle of the micro‐organism. For example, bacteria possess fast rates of reproduction and an ability to horizontally transfer genes,^[^ ^9^ ^]^ which is accelerated when different species are in close proximity to one another, such as those responsible for biofilm infections.^[^ ^10^, ^11^ ^]^ Biofilms refer to communities of micro‐organisms which adhere to a surface and are contained within a self‐produced protective matrix.^[^ ^10^, ^12^ ^]^ This matrix is primarily composed of extracellular polymeric substances (EPS), as well as some additional components such as proteins, nucleic acids, and environmental debris, which provides a protective barrier against factors in the surrounding environment, including antimicrobial agents, such as antibiotics.^[^ ^10^, ^11^, ^12^ ^]^ As such, effective treatment of biofilms requires additional strategies which often include removal of the infected surface (i.e., medical implant) or breaking up the protective matrix.^[^ ^13^ ^]^ Stimuli‐activated antibiofilm treatments will be a particular focus of Section 7.2 of this review. Widespread scientific consensus has concluded that the human overuse and mismanagement of antimicrobial agents has contributed to the rapid development of microbial resistance in pathogenic micro‐organisms.^[^ ^1^, ^14^ ^]^ Notably, as antibiotics become increasingly ineffective, the human population is set to lose its most successful tool, the medical “silver bullet,” returning us to a pre‐antibiotics era, where minor cuts, grazes, and other sources of infection, including routine surgeries, could potentially be fatal.

In addition, fungal infections are also of great concern, particularly in the form of hospital‐acquired infections.^[^ ^15^ ^]^ Fungal infections can result in morbidity and mortality, most notably in immunocompromised patients such as those suffering from AIDS.^[^ ^16^ ^]^ As with bacteria, many fungi can adhere to biotic and abiotic surfaces, presenting as a challenge for removal of the biofilm communities.^[^ ^17^, ^18^ ^]^ For example, *Candida* species were found to be the fourth most common pathogen causing hospital‐acquired bloodstream infection in the USA,^[^ ^19^ ^]^ with around 400 000 cases a year worldwide, which are often associated with implanted medical devices.^[^ ^20^ ^]^ Antifungal drugs predominately target the disruption of the biosynthesis pathways or integrity of important components of the fungal cell wall.^[^ ^21^, ^22^ ^]^ For example, echinocandins prevent the correct synthesis of β‐glucans,^[^ ^23^ ^]^ polyenes bind to membrane sterols,^[^ ^24^ ^]^ while azoles and allylamines inhibit the essential steps in the synthesis pathway of ergosterol.^[^ ^25^ ^]^ Fungal resistance to drugs, however, is a major problem, which is exacerbated by the overuse of antifungal agents in medical contexts, as well as environmental settings, such as the overuse in antifouling coatings and livestock feed formulations.^[^ ^17^, ^26^, ^27^ ^]^ Mechanisms of resistance against antifungal agents vary greatly between and within individual classes of antifungal agents, however these modes of resistance are not as comprehensively understood when compared to bacterial modes of resistance.^[^ ^22^, ^27^, ^28^ ^]^ For example, resistance to the azole class of antifungal agents can be due to the increased activity of specific drug efflux pumps,^[^ ^29^ ^]^ alterations to the enzyme target,^[^ ^30^ ^]^ overexpression of the target enzyme^[^ ^31^ ^]^ and through biosynthesis bypass pathways.^[^ ^32^ ^]^ While resistance to antifungal agents within the polyene class is considered quite rare, resistance has been detected among *Candida* and *Aspergillus* species. The mode of resistance is believed to be caused by inducing a decrease in ergosterol production, which is typically supplemented by an overexpression of other sterols.^[^ ^33^ ^]^ Fungal resistance to echinocandins is poorly understood, however, it is thought to be caused by point mutations in the β‐glucan synthase complex.^[^ ^34^ ^]^ As is the case for bacterial pathogens, multidrug resistance is also an emerging issue in fungal pathogens.^[^ ^35^ ^]^

There is a critical need for the development of new antimicrobial technologies as alternatives to, or to work in combination with, conventional antimicrobial treatment methods.^[^ ^2^ ^]^ There are a range of criteria to which new antimicrobial technologies must conform to in order to be effective. The key criteria include: 1) effective antimicrobial performance, 2) selectivity towards the pathogenic micro‐organism, 3) fast acting, 4) permit clinically practical methods of delivery, 5) low to zero cytotoxicity or other detrimental side effects, and 6) the ability to control the temporal and spatial delivery. The use of metal nanomaterials for their antimicrobial properties has already been shown to address many of these criteria, with varying success.^[^ ^36^, ^37^, ^38^, ^39^ ^]^ In particular, metal nanomaterials have been studied extensively as they possess a range of innate antimicrobial mechanisms, including the disruption of the cellular membrane, diffusion into and degradation of internal cellular components such as DNA, RNA, and enzymes, and the release of ions with antimicrobial activity.^[^ ^40^, ^41^, ^42^ ^]^ Common materials include, but are not limited to: silver, gold, copper, zinc, and their corresponding oxides, with a range of shapes and sizes (typically below 100 nm).^[^ ^37^, ^43^, ^44^, ^45^ ^]^ While there still remains a need for systematic studies to comprehensively explain the bactericidal and fungicidal mechanisms associated with metal nanomaterials, it is clear that they often simultaneously demonstrate several antimicrobial mechanisms.^[^ ^41^, ^46^ ^]^ Therefore, for pathogenic bacteria or fungi to develop resistance they would need to acquire a suite of mutations to counteract the different mechanisms taking place, which is more unlikely than in the case of antibiotics which typically possess a single mode of action.

Next‐generation nanomaterials that can be activated by an external stimulus to illicit antimicrobial properties represent an exciting new step in progress towards an alternative for traditional antimicrobial drugs. Often, the antimicrobial property of the nanomaterial is also responsible for the associated side effects, such as dissolved ions.^[^ ^47^ ^]^ Stimuli‐activated nanomaterials can, however, remain “dormant” until selectively “switched on,” reducing the possibility of detrimental side effects on human cells or beneficial micro‐organisms. Additional benefits include the improved control of the treatment temporally and/or spatially, which enables increased levels of treatment specificity toward the infection region and causative pathogenic micro‐organism. Light and magnetism are the two primary stimuli for current stimuli‐activated antimicrobial nanomaterials, with different mechanisms of action being responsible in each case. Photocatalytic and photothermal nanomaterials are stimulated by the input of energy from certain wavelengths of light to produce reactive oxygen species (ROS) and localized increases in temperature, respectively, which have been observed to be effective against pathogenic bacteria and fungi. Magnetic hyperthermia and magnetophysical nanomaterials respond to magnetic fields to kill pathogens through a localized temperature increase and physical rupture, respectively. In addition to these four stimuli‐activated antimicrobial nanomaterials, there are multiple drug delivery systems that can be activated by a wide range of stimuli,^[^ ^39^, ^48^ ^]^ such as light,^[^ ^49^ ^]^ magnetism,^[^ ^50^ ^]^ ultrasound,^[^ ^51^ ^]^ pH,^[^ ^52^ ^]^ and enzymatic activity;^[^ ^53^ ^]^ however these are beyond the scope of this review which primarily focuses on metal nanomaterials which themselves possess antimicrobial properties as opposed to a mechanism of delivery.

This review focuses on metal nanomaterials, which demonstrate antimicrobial activity. We provide a broad overview of the properties and synthesis of nanomaterials and their passive interactions with bacteria and fungi. Furthermore, we provide an in‐depth analysis and comparison of the next‐generation approaches of stimuli‐activated antimicrobial nanomaterials, providing scope for the design of future antimicrobial treatments.

## 2 Structure of the Bacterial and Fungal Cell Wall

**Figure 1:**
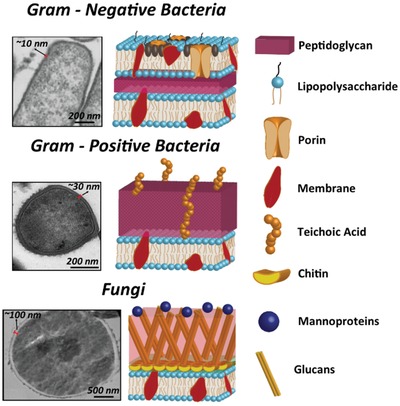
Transmission electron micrographs (left) and schematic diagram (right) of the cell wall of Gram‐negative bacteria (top), Gram‐positive bacteria (middle) and fungi (bottom) cells, respectively. It should be noted that the precise composition of fungal cells can be widely variant amongst species. As such, the schematic is a generalized version of a fungal cell wall. The pictorial legend to the right of the figure provides detail of the cell wall components. Bacterial electron micrographs reproduced with permission.^[^ ^56^ ^]^ Copyright 2019, Elsevier. The fungal electron micrograph reproduced with permission.^[^ ^65^ ^]^ Copyright 2018, Dovepress.

The cell wall is the protective barrier that isolates the internal components of the cell from the external environment. As such, it is immensely important to the cells' ability to survive and flourish.^[^ ^54^ ^]^ For bacteria, the cell wall is a complex structure, composed of proteins, lipids, and carbohydrates, of which there are two primary classes: 1) Gram‐positive and 2) Gram‐negative.^[^ ^55^ ^]^ The Gram‐positive cell wall consists of a thick peptidoglycan layer, surrounding the lipid bilayer membrane with lipoteichoic acids linking the two layers^[^ ^56^, ^57^, ^58^ ^]^ (**Figure** **1**). This thick peptidoglycan layer consists of chains of the alternating disaccharides *N*‐acetylmuramic acid and *N*‐acetylglucosamine, which are connected via β‐1,4 linkages, with individual layers connected by pentapeptide cross‐links to form a think, robust layer. This provides the cell with enhanced levels of protection from external chemical and physical factors.^[^ ^54^, ^59^ ^]^ Conversely, the Gram‐negative cell wall is more complex, possessing a thinner peptidoglycan layer than Gram‐positive bacterium, which is sandwiched between an inner and outer cell membrane; the latter consisting of negatively charged lipopolysaccharides^[^ ^54^, ^60^ ^]^ (Figure 1). This external membrane is unique to Gram‐negative bacterium and acts as the interface between the environment and the cell, and is largely responsible for protection as well as contributing to the cells pathogenicity, namely the presence of lipopolysaccharides.^[^ ^61^ ^]^ Additional components in the outer membrane include porins, which allow the diffusion of molecules that would not be able to otherwise translocate through the cell membrane, lipoproteins, periplasmic space and numerous membrane‐bound proteins that serve specific functions for the cell.^[^ ^58^ ^]^ Due to the complex nature of the bacterial cell wall and the fundamentally dynamic nature of the cell as a living organism, interactions between nanomaterials and the cell wall are still not well understood and are thought to consist of a combination of physicochemical forces such as electrostatic, hydrodynamic, hydrophobic, dispersion, and van der Waals forces.^[^ ^56^, ^62^ ^]^

Conversely, fungi are eukaryotic cells. They are nucleated and possess a distinctly different cell wall to that of bacteria. The fungal cell surface consists of a phospholipid bilayer membrane, linked with unbranched chains of polymers known as chitin, which are cross‐linked to β‐1,3‐ and β‐1‐6‐glucans, as well as additional membrane‐bound proteins, which serve a range of functions^[^ ^63^ ^]^ (Figure 1). While this is the base structure of the fungal cell wall, many fungi have additional components such as mannan (*Candida albicans*), melanin (*Aspergillus fumigatus*), glucuronoxylomannan and galactoxylomannan (*Cryptococcus*), as well as other components, which can influence the properties of the cell wall and are often used for specific functions.^[^ ^64^ ^]^

## 3 Fundamental Aspects of Metal Nanomaterials

### 3.1 Properties

**Figure 2:**
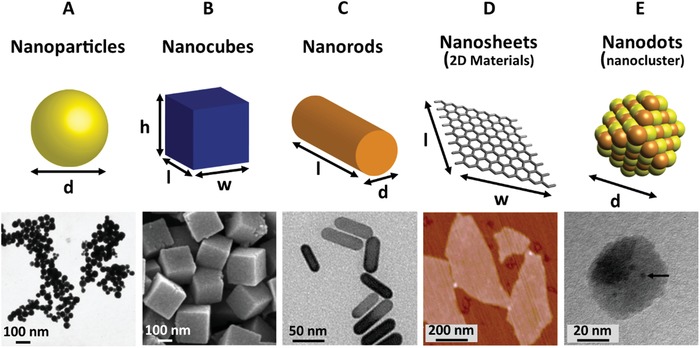
Schematic (top) and experimental (bottom) representation of the range of different nanomaterials that possess passive antimicrobial activity. Experimental data (bottom row) shows A) TEM image of silver nanoparticles. Reproduced with permission.^[^ ^74^ ^]^ Copyright 2006, ACS Publications. B) SEM image of ZnSn(OH)_6_ nanocubes. Reproduced with permission.^[^ ^76^ ^]^ Copyright 2012, ACS Publications. C) TEM image of gold/silver hybrid nanorods. Reproduced with permission.^[^ ^78^ ^]^ Copyright 2018, ACS Publications. D) AFM image of graphene nanosheets. Reproduced with permission.^[^ ^86^ ^]^ Copyright 2011, ACS Publications. E) TEM image of copper nanodots (clusters). Reproduced with permission.^[^ ^75^ ^]^ Copyright 2019, ACS Publications.

At the nanoscale, the physical and chemical properties of metals change dramatically from that of the bulk material.^[^ ^66^ ^]^ This is primarily due to size and shape effects, as well as the high surface area to volume ratio inherent to nanomaterials.^[^ ^66^ ^]^ Importantly, this results in changes to the fundamental properties of the nanomaterial, such as expedited ion release,^[^ ^67^ ^]^ hardness,^[^ ^68^ ^]^ and plasmonic and superparamagnetic properties.^[^ ^69^ ^]^ Metal nanomaterials respond differently to external stimuli, such as light in the case of photocatalytic^[^ ^70^ ^]^ and photothermal^[^ ^71^ ^]^ activity and magnetism in the case of magnetically induced hyperthermia activity,^[^ ^71^, ^72^ ^]^ in contrast to their bulk‐metal counterparts. In addition to size, the shape of the nanoparticles can also influence their intrinsic properties, for example, photocatalytic properties can be affected, largely through enhanced surface area while plasmonic properties are influenced by the nanomaterials shape which affects the relative lengths along which the electron cloud can resonate and hence the specific wave function.^[^ ^73^ ^]^ With improvements in nanotechnology and fabrication processes, a diverse array of nanoscale shapes have been constructed, such as: nanoparticles,^[^ ^74^ ^]^ nanodots,^[^ ^75^ ^]^ nanocubes,^[^ ^76^, ^77^ ^]^ nanorods,^[^ ^78^, ^79^ ^]^ nanoshells,^[^ ^80^ ^]^ nanocages,^[^ ^81^ ^]^ nanostars,^[^ ^82^ ^]^ nanoflowers,^[^ ^83^ ^]^ nanoeggs,^[^ ^84^ ^]^ nanopopcorn,^[^ ^85^ ^]^ and numerous other 2D materials.^[^ ^86^, ^87^ ^]^ **Figure** **2** shows a variety of commonly investigated nanomaterials, along with experimentally obtained images of example nanomaterials with the corresponding shape. The unique properties of metals at the nanoscale have led to research into the use of these nanomaterials for different applications, ranging from next‐generation electronics to numerous biomedical applications.

### 3.2 Synthesis

**Figure 3:**
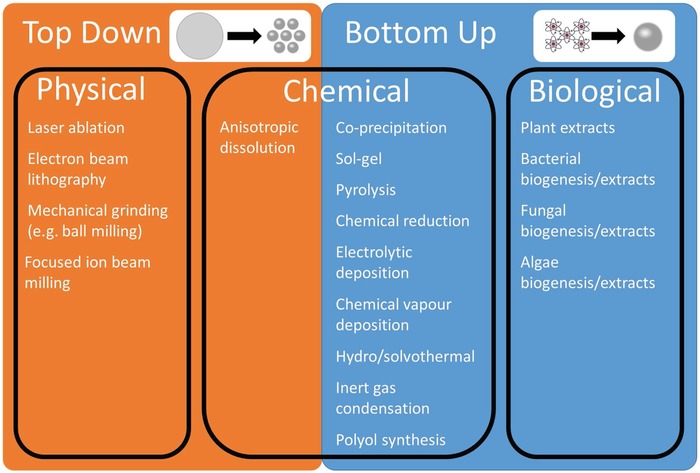
Summary of “top down” and “bottom up” physical, chemical and biological synthesis of metal nanomaterials.

The synthesis of nanomaterials can largely be categorized into two methods: “top down”^[^ ^88^, ^89^ ^]^ or “bottom up”^[^ ^90^, ^91^ ^]^ processes (**Figure** **3**). For the former, particles are generally fabricated via the breaking down of bulk materials into smaller (nano‐) fragments, typically by physical means.^[^ ^88^, ^92^ ^]^ This includes methods such as laser ablation, electron beam lithography, mechanical grinding, or focused ion beam milling. For example, Ismail et al. synthesized magnetic iron oxide nanoparticles through a process of laser ablation in solution, within the 50–110 nm range, which demonstrated antibacterial activity against Gram‐positive and Gram‐negative bacterium.^[^ ^93^ ^]^ Whereas, for “bottom up” approaches, particles are often chemically grown from precursors, through a process of chemical reduction.^[^ ^90^, ^94^ ^]^ Typically, metal cations, in the form of salts, are combined with a reducing agent such as sodium borohydride or sodium citrate and reduced to a neutral state.^[^ ^95^ ^]^ Following an initial nucleation step, the atoms cluster together, forming a seed of defined crystallinity, which then grows larger until the process is interrupted through the addition of a capping agent, the timing of which dramatically affects the size of the nanoparticles.^[^ ^96^ ^]^ Often stabilizing agents, such as ligands or polymers, are added for greater control over the size of the nanomaterials.^[^ ^97^ ^]^ Additionally, there are several other processes such as inert gas condensation,^[^ ^98^ ^]^ sol–gel,^[^ ^99^ ^]^ coprecipitation,^[^ ^100^ ^]^ among others.^[^ ^101^ ^]^ For example, Samavati and Ismail used a process of coprecipitation to synthesize copper‐substituted cobalt ferrite nanoparticles by adding CoCl_2_·6H_2_O, CuCl_2_·2H_2_O and FeCl_3_ together in distilled water, with the addition of citric acid as a chelating agent and NaOH to maintain a pH of 8. The precipitates were annealed at 800 °C for 10 h, forming nanoparticles in diameter within the range of 20–32 nm, which they found to have antibacterial activity against multidrug‐resistant *E. coli*.^[^ ^102^ ^]^ Interestingly, there has been a push for biological methods of nanomaterial synthesis via plants and micro‐organisms,^[^ ^103^, ^104^ ^]^ which avoid using toxic or environmentally damaging chemicals unlike traditional methods. A detailed description of nanomaterial synthesis methods is beyond the scope of this review article; however, the interested reader is directed to several important methodological reviews in the field.^[^ ^90^, ^96^, ^104^ ^]^ A summary of physical, chemical, and biological methods for nanoparticle synthesis is provided in Figure 3.

## 4 Passive Antimicrobial Mechanisms of Metal Nanomaterials

Metals, such as copper and silver, have been utilized for their antimicrobial properties for thousands of years for applications such as water disinfection, food preservation, and surgical bandages, and sutures.^[^ ^46^, ^105^ ^]^ While these materials were not extensively explored in early scientific studies, partly due to the discovery of antibiotics in the 1920s, there has been reinvigorated interest in the use of metal nanomaterials as antimicrobial agents. This renewed interest coincides with significant breakthroughs in the understanding, fabrication and characterization of sub‐micron‐sized materials. A wide range of nanomaterials have been demonstrated to possess antimicrobial effects, including iron (III) oxide,^[^ ^106^ ^]^ zinc oxide,^[^ ^107^ ^]^ magnesium oxide,^[^ ^108^ ^]^ silver,^[^ ^67^, ^109^ ^]^ gold,^[^ ^110^ ^]^ copper^[^ ^45^, ^77^ ^]^ and copper oxide,^[^ ^111^ ^]^ calcium oxide,^[^ ^41^ ^]^ titanium dioxide^[^ ^112^ ^]^ and cadmium oxide^[^ ^113^ ^]^ among others.^[^ ^41^, ^42^, ^87^, ^114^, ^115^ ^]^ Successful antimicrobial activity has been demonstrated utilizing an equally wide array of shapes (Figure 2).

**Figure 4:**
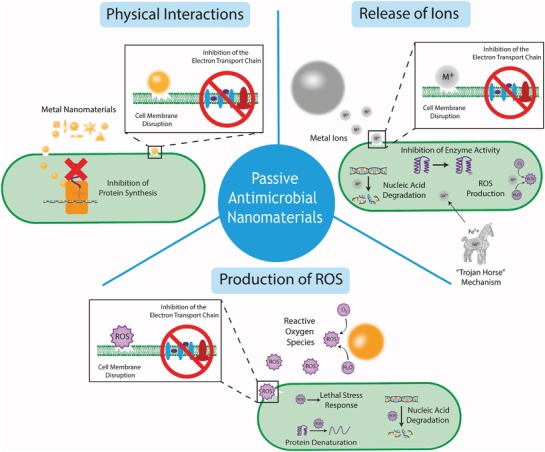
A summary schematic diagram representing the range of passive antimicrobial mechanisms of metal nanomaterials (not to scale) including physical interactions, release of ions and production of ROS.

Despite several explanations for nanomaterial–microbial interactions, the mechanisms responsible for the passive antimicrobial properties of metal nanomaterials are still poorly understood. This is partly due to the multi‐factorial nature of the activity, which makes it difficult to decouple the individual mechanisms. Several different proposed mechanisms were derived from physical interactions as well as chemical interactions, such as the production of ROS and the increased dissolution of metal cations^[^ ^37^, ^41^, ^43^, ^116^, ^117^, ^118^ ^]^ (**Figure** **4**). These mechanisms can have numerous target sites, such as the cell membrane, membrane‐bound proteins, inhibition of enzyme activity and nucleic acids, hence it is proposed that it is more difficult for pathogenic bacteria and fungi to develop resistance to protect all of these cellular components.^[^ ^38^ ^]^ Due to the lack of clarity within the current literature, we have divided such proposed mechanisms into three broad categories 1) physical interactions, 2) ion leaching/dissolution, and 3) production of ROS. It is noteworthy that within these categories remains overlaps (for example metal cations can increase intracellular ROS), however broadly these cover the primary modes of passive antimicrobial activity of metal nanomaterials. Information regarding the inherent difference in bacterial and fungal cell interactions with antimicrobial nanomaterials are rarely reported in isolation. Instead, the known antibacterial mechanisms of metal nanomaterials are often directly attributed to antifungal behavior, without discussion of known differences between the microbes, such as their cell walls (Figure 1). Hence the majority of literature reporting on the antifungal activity of metal nanomaterials conflates antibacterial behavior with both antifungal and antimicrobial. Careful review of the literature, therefore, reveals no clear consensus regarding the precise mechanisms of antifungal and/or antimicrobial activity of metal nanomaterials. To this end, the authors of this review suggest that elucidating the specific antifungal mechanisms of metal nanomaterials should be the focus of future studies. For more comprehensive reviews on the passive antimicrobial activity of metal nanomaterials, readers are encouraged to read several comprehensive reviews on this topic.^[^ ^46^, ^105^, ^118^ ^]^

### 4.1 Physical Interactions

In this section, we summarize the direct activity of metal nanomaterials due to physical interactions (i.e., not through the generation of a subsequent chemical species such as ion release or ROS which will be summarized below). These interactions can be further divided into two primary sections: 1) interactions with the cell wall and 2) intracellular activity.

The cell wall presents as the barrier between the cell and its external environment, as well as facilitating important metabolic pathways (e.g., the electron transport chain) and hence disruption of the cell wall can be fatal for the cell. Metal nanomaterials, which are typically positively charged, can bind to the negatively charged cell wall components through electrostatic interactions, which alter the charge of the membrane, disrupting the membrane,^[^ ^105^, ^118^ ^]^ as well as forming ROS species. Damage to the membrane causes significant leakage of fluid from the cytosol as well as irreparable damage to the cell wall and ATP production, effectively inactivating the cells.^[^ ^105^ ^]^ It is worth noting that the influence on the antimicrobial activity of nanomaterial induced cell membrane damage through physical interactions can be overreported as cell membrane damage can often be the result of another biocidal mechanism (such as an increase in intracellular ROS) rather than the primary cause of cell death. Furthermore, because membrane damage is uncomplicated to visualize, typically through electron microscopy techniques and/or the use of membrane‐permeable or membrane‐impermeable dyes and fluorescent or confocal laser scanning microscopy, it can be easily over‐attributed as the cause of cell death.

Cellular uptake of metal nanomaterials can occur when the materials are sufficiently small that they can cross the cellular membrane. In the case of mammalian cells, it has been suggested that particles below 100 nm are most efficient for cellular uptake.^[^ ^119^ ^]^ However due to the multiple mechanisms of cellular uptake present in mammalian cells, they can also internalize larger particles. Furthermore, the surface chemistry of the particles also plays an important role in particle uptake, which can be modulated through the addition of different surface coatings.^[^ ^120^ ^]^ While there is significant research relating to cellular uptake in mammalian cells, the cellular uptake pathways in bacteria and fungi are less well studied. One study demonstrates the internalization of ZnO and TiO_2_ nanoparticles of 30 and 50 nm, respectively, as measured by TEM, in *Salmonella typhimurium*.^[^ ^121^ ^]^ Once internalized, metal nanomaterials can interact with important cellular components, for example, gold nanoparticles have been shown to bind to the ribosomal subunit, which inhibits successful binding of tRNA,^[^ ^116^ ^]^ which serves an important role in successful protein synthesis.^[^ ^122^ ^]^ However, the research is not yet clear as to the specific components that are affected and how this changes through the use of different metals, partly due to difficulties in visualizing these interactions experimentally. Future research will require carefully planned studies using a combination of high‐resolution imaging and metabolic/genomic studies to decouple the effects of the physical (such as binding to proteins/nucleic acids) and the chemical (release of ions and production of ROS) interactions of internalized metal nanomaterials, to gain a better understanding of these mechanisms.

### 4.2 Ion Leaching/Dissolution

Metal nanomaterials leach metal ions at a much higher rate than the bulk material, due to the significantly enhanced surface area, when compared to equivalent volumes of the bulk. These ions can detrimentally interact with various cellular components such as proteins, DNA, and the cellular membrane.^[^ ^46^, ^74^, ^123^ ^]^ Ions can be taken up by bacteria and fungi through a suite of transport proteins, which control the uptake of metal species.^[^ ^124^ ^]^ Metal ions have been shown to demonstrate multiple antimicrobial mechanisms, which often occur in a synergistic manner and researchers have faced significant challenges elucidating the individual mechanisms, however the current theories are detailed below. 1) Inhibition of enzyme activity, which can occur via metal‐catalyzed oxidation of amino acid residues in proteins.^[^ ^46^ ^]^ 2) Generation of ROS, either directly in the case of redox‐active metals, or through damage to the Fe–S clusters within proteins, which liberate redox‐active Fe ions.^[^ ^46^ ^]^ 3) Inhibition of nutrient uptake, for example gallium ions have recently been shown to kill bacteria through a “trojan horse” mechanism, whereby the cells mistake it for Fe^3+^ ions, due to the similar chemical properties^[^ ^125^ ^]^ and the cell becomes inactivated through inhibition of metabolic activity as the bacteria are unable to reduce the Ga^3+^.^[^ ^126^ ^]^ 4) Damage to the membrane can occur as the positive metal cations interact with the electronegative membrane as well as some integral proteins such as those involved in the electron transport chain.^[^ ^46^ ^]^ Furthermore, damage to DNA has also been shown, demonstrating the genotoxic activity of metal ions, but it is not established whether this is a primary cause of cell death in vivo.^[^ ^46^ ^]^ The most prevalent examples are silver ions, which have been shown to interact with cell membranes, nucleic acids, and the thiol and amino groups which are present in proteins, with bactericidal^[^ ^67^, ^74^, ^127^ ^]^ and fungicidal^[^ ^128^ ^]^ effects. Unfortunately, it is thought that microbial pathogens will eventually develop resistance to nanosilver.^[^ ^129^ ^]^ Other metals, such as copper, have also been shown to leach ions which exhibit antimicrobial activity.^[^ ^123^ ^]^ The primary drawbacks of this mechanism is the development of resistance mechanisms, such as the overexpression of efflux pumps, as well as associated side effects of ion dissolution, which have been shown to possess cytotoxic properties.^[^ ^47^ ^]^ Ion dissolution will remain an important concept and consideration for the future designs of antimicrobial metal nanomaterials, which will ideally work in combination with an additional antimicrobial mechanism to reduce the prospect of the development of pathogen resistance.

### 4.3 Production of Reactive Oxide Species

Reactive oxygen species (ROS), which include singlet oxygen (^1^O_2_), superoxide anion radicals (^•^O_2_^–^), hydroxyl radicals (^•^OH) and hydrogen peroxide (H_2_O_2_) are produced endogenously in the cell through natural processes. Under normal circumstances, the cell is able to function in the presence of low levels of ROS through repairing mechanisms for damaged cell components^[^ ^130^ ^]^ and naturally produced ROS scavenging enzymes,^[^ ^131^ ^]^ which protect the cells from the associated oxidative stress. In higher concentrations, however, ROS cause oxidative stress on bacterial cells, which can cause significant damage to the cell membrane,^[^ ^132^, ^133^ ^]^ degrade important proteins and nucleic acids^[^ ^130^, ^134^ ^]^ and initiate lethal stress response cascades,^[^ ^131^ ^]^ ultimately leading to cell death. Similarly, the overproduction of ROS can have antifungal effects.^[^ ^135^ ^]^ Metal nanomaterials can induce cells to increase the generation of ROS through metabolic responses, through the promotion or suppression of ROS‐related enzymes.^[^ ^136^ ^]^ Furthermore, metal nanomaterials can directly participate in ROS generation, for example, Lipovsky et al. demonstrated that the antifungal effects of ZnO nanoparticles against *Candida albicans* were significantly reduced through the addition of histidine, a molecule which quenches hydroxyl radicals and singlet oxygen, hence concluding the primary antifungal effect was achieved through the exogenous production of ROS by the nanoparticles.^[^ ^137^ ^]^ Further, there has been extensive screening of the antibacterial activity of ROS‐producing metal nanomaterials, including silver,^[^ ^138^, ^139^ ^]^ zinc oxide,^[^ ^140^ ^]^ and titanium dioxide,^[^ ^132^ ^]^ among others. The amount of ROS generated from metal nanomaterials is primarily dependent on the size^[^ ^139^, ^141^ ^]^ and chemistry^[^ ^142^ ^]^ of the nanomaterial, while the effect of the shape of the particle is less well known.

## 5 Molecular Modeling to Enhance Antimicrobial Nanomaterial Development

While the exact mechanism of antimicrobial action for many nanomaterial treatments is poorly understood, molecular modeling has shown the potential to inform the development of future methods by describing the key interactions between materials and microbes. Molecular modeling techniques relevant to metal nanomaterial development utilize theoretical approaches and algorithms to relate the 3D structure of molecules and materials to their behavior and properties, and include molecular dynamics (MD) simulations, quantum mechanics (QM) calculations, and machine learning (ML) methods.

In classical atomistic MD simulations, molecules are typically represented by atomic beads with a fixed charge that are connected by bonds, angles, and dihedrals, while intermolecular interactions are described by electrostatic and van der Waals terms. While this simplistic treatment allows for simulations of up to billions of atoms^[^ ^143^ ^]^ and up to millisecond timescales,^[^ ^144^ ^]^ because bonds and atomic partial charges are fixed, chemical reactions, optical or electronic properties, and the effects of polarization cannot be thoroughly examined. Nevertheless, MD methods have proven useful in studying the bacterial cell wall,^[^ ^145^ ^]^ elucidating the process of fungal biofouling^[^ ^146^ ^]^ and providing insight into antifouling materials,^[^ ^147^ ^]^ and by providing design principles for photoluminescent nanoparticles,^[^ ^148^ ^]^ among others. In order to provide atomistic insight into the mechanism of membrane permeation and disruption, however, accurate models of the microbial cell membrane and cell wall are essential. Computational models of realistic cell membranes have been recently extensively reviewed,^[^ ^149^ ^]^ and a web‐based interface for the construction of lipopolysaccharides found in Gram‐negative bacterial cell walls has been developed.^[^ ^150^ ^]^ This can aid in the modeling of bacterial membranes even for inexperienced users. While several models of the bacterial peptidoglycan layer have been developed,^[^ ^151^ ^]^ including the use of an atomistic reactive force field to model plasma‐induced destruction,^[^ ^152^ ^]^ these models are often not compatible with the model of the nanomaterial of interest. For example, a 2016 review highlighted the challenges and achievements of modeling gold nanoparticles and materials at biological interfaces.^[^ ^153^ ^]^ It is therefore critical that accurate, compatible models for relevant microbial components and models of relevant nanomaterials, ions, and ROS molecules be obtained. Once this is achieved, the effects of metal nanomaterials on bacterial and fungal cell walls and membranes, ion and ROS interactions, and passive antimicrobial activity of nanomaterials should be open avenues for investigation via MD‐based computational methods. Indeed, a recent paper describes the use of a reactive force field to model the ROS‐induced destruction of the fungal cell wall at the atomic level.^[^ ^154^ ^]^

QM calculations and ML methods are also available to guide the development of stimuli‐activated antimicrobial nanomaterials by providing information on bandgaps,^[^ ^155^ ^]^ photothermal,^[^ ^156^ ^]^ photocatalytic,^[^ ^157^ ^]^ and magnetic^[^ ^158^ ^]^ properties. In contrast to classical MD simulations, QM methods, or more specifically density functional theory (DFT), calculate electronic structure explicitly and thus may be used to describe chemical reactions as well as optical and electronic properties. While DFT methods provide greater chemical accuracy, they also require greater computational resources due to the increased complexity, with current upper limits of tens of thousands of atoms^[^ ^159^ ^]^ and nanosecond timescales for ab initio MD.^[^ ^160^ ^]^ ML methods, which require data sets of either experimental or calculated properties, can provide non‐intuitive understanding of structure–property relationships and even predict the values from computationally expensive DFT calculations^[^ ^161^ ^]^ or use data from MD simulations to better understand microbial contamination.^[^ ^162^ ^]^

While a comprehensive overview of all possible computational techniques is outside the scope of this review, we point the reader to a recent review of computational modeling of magnetic nanoparticle properties for further examples.^[^ ^163^ ^]^

## 6 Light‐Activated Antimicrobial Metal Nanomaterials

### 6.1 Photocatalytic Antimicrobial Metal Nanomaterials

#### 6.1.1 Antimicrobial Mechanism of Photocatalytic Metal Nanomaterials

**Figure 5:**
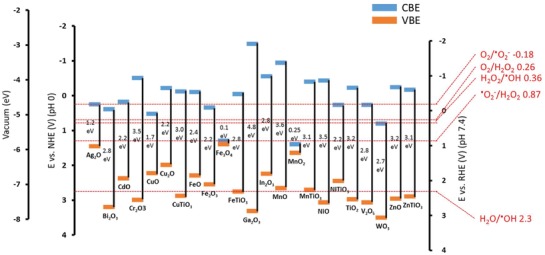
Redox potentials for the generation of reactive oxygen species by semiconductors often used for photocatalytic antimicrobial activity. Includes bandgap, conduction band edge (CBE), and valence band edge (VBE) in relation to vacuum scale in electron volts (eV), normal hydrogen electrode (NHE pH 0) and reversible hydrogen electrode (RHE pH 7.4) in volts (V). Values are reproduced with permission.^[^ ^169^ ^]^

Photocatalytic nanomaterials can be activated by light to produce free ROS, which have associated antimicrobial properties.^[^ ^41^, ^70^, ^112^, ^164^, ^165^, ^166^ ^]^ Typically these nanomaterials are made from semiconductor materials, which have a relatively small difference between the valence band and conduction band, known as the energy “bandgap.”^[^ ^167^ ^]^ The valence band of an atom is the outer‐most orbital that electrons freely occupy when the material is in its ground state; meanwhile, the conduction band describes the higher energy orbitals into which electrons can freely transition when the material is in an excited state. When electrons are in the conduction band, they possess sufficient energy to move freely in the material, resulting in conductivity. In conducting materials, these bands are overlapped, hence the material is permanently conductive. Conversely, in insulating materials the bandgap becomes too large for the electrons to move into the conduction band (or requires an unreasonable amount of energy); hence, the material is insulating. Importantly, in some semiconductor materials, the gap between these two bands is sufficiently small that the input of energy from certain bandwidths on the electromagnetic spectrum can cause electrons to transition from the valence to conduction band (**Figure** **5**).^[^ ^168^ ^]^ The bandgap distance is dependent on the electron configuration of the material and hence the required energy input (in the form of light) is significantly influenced by the composition of the semiconductor.

**Figure 6:**
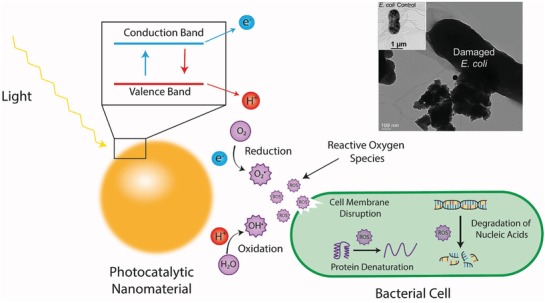
Schematic representation of the photocatalytic effect of metal nanomaterials and subsequent antimicrobial mechanism. The energy input from light results in a free electron in the conduction band and an electron hole in the valence band which react with O_2_ and H_2_O, respectively, to form reactive oxygen species, which exhibit a number of antimicrobial mechanisms. Top right is a transmission electron micrograph of *E. coli* cells exposed to photocatalytic nanoparticles, which demonstrates significant membrane damage compared to the control (inset). Reproduced with permission.^[^ ^171^ ^]^ Copyright 2013, Royal Society of Chemistry.

When an electron makes this transition from the valence to the conduction band, a hole is left in the valence band and the conduction band gains a free electron. This induced state leads to one of, or a combination of, two actions: 1) the electrons can instantaneously recombine, resulting in energy being released in the form of heat or radiation 2) the free electron and electron hole can react with electron acceptors and electron donors, which come into contact with the surface of the semiconductor.^[^ ^164^, ^170^ ^]^ The last case is true for photocatalytic nanomaterials with antimicrobial properties (**Figure** **6**).

Typically in these cases, the electron acceptor is O_2_ and the electron donor is H_2_O. The free electron is capable of reducing O_2_ to the superoxide ion ^•^O_2_^−^ which can be oxidized to form singlet oxygen ^1^O_2_ or act as a precursor for the hydroxyl radical ^•^OH and hydrogen peroxide H_2_O_2_ which are powerful oxidizing agents. Simultaneously, the electron hole is capable of oxidizing H_2_O to form ^•^OH which can dimerize to form H_2_O_2_. A summary of these reactions and the required redox potentials at physiological pH are shown in **Table** **1** and referenced in Figure 5.

**Table 1:** Redox potentials for the generation of reactive oxygen species (ROS)

Half‐reaction	Redox couple	Redox potential versus RHE at physiological pH 7.4 [V]^a)^
O_2_ + → e^−^ + ^•^O_2_^−^	O_2_/^•^O_2_^−^	−0.18
^•^O_2_^−^ + 2H^+^ + e^−^ → H_2_O_2_	^•^O_2_^−^/H_2_O_2_	0.87
H_2_O_2_ + H^+^ + e^−^ → H_2_O + ^•^OH	H_2_O_2_/^•^OH	0.36
H_2_O + H^+^ → 2H^+^ + ^•^OH	H_2_O/^•^OH	2.30
O_2_ + 2H^+^ + 2e^−^ → H_2_O_2_	O_2_/H_2_O_2_	0.26

a) Redox potentials were calculated using the method described in reference ^[^ ^172^ ^]^ and the Nernst Equation.

The produced ^•^O_2_^−^, ^•^OH radicals, and H_2_O_2_ are the key to the antimicrobial properties of photocatalytic nanomaterials. Additionally, the production of singlet oxygen (^1^O_2_), which is a strong oxidation reagent, has also been demonstrated through photocatalysis with metal nanomaterials.^[^ ^173^ ^]^ The antimicrobial activity of these ROS has not been conclusively determined, however, it is suggested that there are a variety of mechanisms.^[^ ^164^ ^]^ It is hypothesized that the ROS first interact with the bacterial membrane, where they can cause oxidative damage, disrupting the cell wall, which exposes the intracellular compartment of the cell to its external environment. This action leads to an uncontrolled movement of components in and out of the cell, and eventually cell death.^[^ ^170^, ^174^ ^]^ Furthermore, ROS have been shown to damage several intracellular components of the cell, such as important nucleic acids, lipids, and proteins, which may increase the speed and efficacy of the antibacterial activity.^[^ ^175^, ^176^ ^]^ Because the generation of ROS occurs at the interface of the material and surrounding fluid, nanomaterials with smaller sizes or high aspect ratio morphologies generally possess improved antimicrobial efficacy as the specific surface area of the material is greatly increased.^[^ ^177^, ^178^ ^]^

#### 6.1.2 Antimicrobial Activity of Photocatalytic Metal Nanomaterials

The antimicrobial activity of photocatalytic metal nanomaterials was first demonstrated by Matsunaga et al. using a powder consisting of the semiconductor TiO_2_ and Pt (9:1 wt%).^[^ ^179^ ^]^ These authors demonstrated the photochemical sterilization of the Gram‐negative bacteria *Escherichia coli* and *Lactobacillus acidophilus*, as well as *Saccharomyces cerevisiae* (yeast) and *Chlorella vulgaris* (algae). The powder was incubated with each micro‐organism in solution and the antimicrobial activity was assessed by plate counts of serially diluted solutions at different treatment time points. Following a 120 min exposure to light in the UV–vis spectrum, produced by a metal halide lamp, they observed complete sterilization of *E. coli* and *L. acidophilus*.^[^ ^179^ ^]^ Following this initial study, there have been many significant improvements in the development of photocatalytic nanoparticles, such as greater control over the wavelength of light, which induces photocatalysis, successful conjugation of biomolecules such as antibodies and aptamers for more targeted treatments and a broadening of effective materials beyond TiO_2_ assessed against a broad spectrum of Gram‐negative and Gram‐positive bacteria, as well as a range of fungal species.

The photocatalytic properties of TiO_2_ occur under UV light, which provides sufficient energy to cause the electrons to jump from the valence to conduction band (bandgap ≈3.2 eV). The use of UV light is manageable for external use, such as sterilization of surfaces, however the use of UV is not practical in clinical situations as it poses a threat to human cells and the high energy input required is not very efficient.^[^ ^180^ ^]^ Recently, research in the field has been focused on reducing the energy bandgap to enable catalytic activity under irradiation by visible light (≈400–700 nm) and near infrared (NIR, ≈750–2500 nm). To be effective in deep tissue infections, the excitation wavelength ideally needs to be shifted into the NIR range, which is able to penetrate through human tissue, often termed the “biological window,” which have been previously reported as occurring at 650 ≤ λ ≤ 950 nm and 1000 ≤ λ ≤ 1350 nm.^[^ ^181^ ^]^ This can be achieved through the use of different materials, or doping TiO_2_ with other transition metals, providing secondary energy levels closer to the TiO_2_ conduction band.^[^ ^182^, ^183^, ^184^ ^]^ This reduces the bandgap energy required for the electrons to jump between the two bands, and hence a lower input of energy (such as from visible or NIR light) is required for photocatalytic activity. An example of conduction and valence band edges as well as bandgaps from a variety of metal materials is provided in Figure 5. For example, Yadav et al. demonstrated the significant photocatalytic inactivation of both Gram‐positive and Gram‐negative bacteria using nickel‐doped TiO_2_ nanoparticles, through a sol‐gel method, under low intensity (≈0.5 mW cm^−2^) visible light (>400 nm) irradiation.^[^ ^183^ ^]^ Additionally, antimicrobial activity has been observed under visible light irradiation with TiO_2_ nanoparticles doped with copper,^[^ ^182^, ^184^ ^]^ graphene,^[^ ^185^ ^]^ silver,^[^ ^186^ ^]^ silver and nitrogen,^[^ ^187^ ^]^ sulfur^[^ ^188^ ^]^ and cadmium sulfide.^[^ ^189^ ^]^ Understanding these principles will provide important design parameters for next‐generation, light‐stimuli responsive antimicrobial nanomaterials.

**Figure 7:**
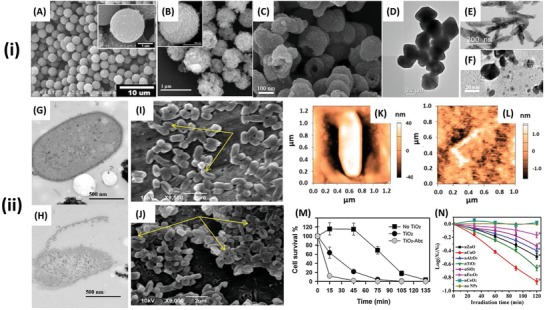
i‐A–C) SEM and D–F) TEM images of antibacterial, photocatalytic nanomaterials A) TiO_2_ conjugated with an *E. coli* specific antibody. Reproduced with permission.^[^ ^176^ ^]^ Copyright 2012, ACS Publications. B) CdIn_2_S_4_ microspheres. Reproduced with permission.^[^ ^190^ ^]^ Copyright 2013, Elsevier. C) Porous fluorinated SnO_2_ hollow nanospheres. Reproduced with permission.^[^ ^191^ ^]^ Copyright 2014, ACS Publications. D) BiVO_4_ nano‐octahedrals. Reproduced with permission.^[^ ^192^ ^]^ Copyright 2016, Elsevier. E) ZnO nanorods. Reproduced with permission.^[^ ^193^ ^]^ Copyright 2018, MDPI. F) CdS spheroids. Reproduced with permission.^[^ ^189^ ^]^ Copyright 2014, Elsevier. ii) Visualization and quantification of the antibacterial activity of photocatalytic nanomaterials. TEM images of G) *E. coli* before and H) after 6 h treatment with CdIn_2_S_4_ microspheres under visible light irradiation. Reproduced with permission.^[^ ^190^ ^]^ Copyright 2013, Elsevier. SEM images of *E. coli* following 3 h treatment with ZnO nanorods, I) in the dark and J) under light irradiation. The arrows indicate some of the most visibly summarized in Table damaged cells, which are more abundant in the cells exposed to light. Reproduced with permission.^[^ ^193^ ^]^ Copyright 2018, MDPI. AFM images of *E. coli* K) before and L) after 3 h exposure to photocatalytic ZnO nanorods under light irradiation. Reproduced with permission.^[^ ^193^ ^]^ Copyright 2018, MDPI. M) Inactivation of *E. coli* with TiO_2_, TiO_2_ conjugated with an *E. coli* specific antibody (TiO2‐Ab_E_) and control under UV irradiation. Reproduced with permission.^[^ ^194^ ^]^ Copyright 2014, Elsevier. N) Kinetics of *E. coli* inactivation by different types of metal‐oxide NPs under UV irradiation. Asterisks (*) denote a significant difference from the control at the 95% confidence level. Reproduced with permission.^[^ ^176^ ^]^ Copyright 2012, ACS Publications.

In conjunction with TiO_2_, other semiconductor metal oxides with similarly small bandgaps have been investigated for their potential antimicrobial properties^[^ ^114^, ^189^, ^190^, ^191^, ^192^, ^193^, ^194^, ^195^, ^196^, ^197^, ^198^, ^199^, ^200^, ^201^, ^202^ ^]^ (see **Table** **2**). For example, Seven et al. demonstrated a significant reduction in the viability of *E. coli*, *Pseudomonas aeruginosa* and *Staphylococcus aureus* cells in the presence of ZnO nanoparticles under irradiation of a broad‐range UV lamp (250–400 nm).^[^ ^195^ ^]^ Interestingly, Padmavathy and Vijayaraghavan found that antibacterial activity against *E. coli* was improved using smaller particles, likely due to the increased number of ROS producing particles interacting with individual *E. coli* cells.^[^ ^177^ ^]^ Promising results have also been demonstrated when ZnO has been combined with other materials. For example, Zhou et al. report reductions in bacterial viability of 99.45% for *S. aureus* and 95.65% for *E. coli*, respectively using nanohydroxyapatite (n‐HA)/ZnO NP complexes under UV light irradiation.^[^ ^196^ ^]^ Furthermore, Kavitha et al. demonstrated the antibacterial potential of ZnO–graphene composite NPs, where they suggested the 2D graphene induced physical rupture of the bacterial cell wall, providing a complimentary bactericidal mechanism to the photocatalytic activity of the ZnO.^[^ ^203^ ^]^ Liu et al. were able to show antimicrobial photocatalytic activity against *E. coli* using silver phosphate (Ag_3_PO_4_) nanoparticles wrapped in graphene oxide.^[^ ^198^ ^]^ In the visible light range, Singh and co‐workers demonstrated ROS production and associated antibacterial activity against *E. coli*, using CuO nanorods.^[^ ^199^ ^]^ In a comprehensive study, Zhang et al. compared the generation of ROS and subsequent antibacterial activity of a wide range of metal nanoparticles and determined AgNPs were the most effective, followed by SiNPs, NiNPs, and AuNPs in descending order.^[^ ^114^ ^]^ This was partially because AgNPs generate superoxide and hydroxyl free radicals, whereas the other three metal oxides only produce singlet oxygen species. More exotic metals and nanoscale shapes have also been investigated, Sharma et al. were able to demonstrate antimicrobial photocatalytic activity against *E. coli* under visible light irradiation using bismuth vanadate (BiVO4) nano‐octahedrals.^[^ ^192^ ^]^ Examples of the range of nanomaterials used and associated antibacterial activity are listed in Table 2 and can be visualized in **Figure** **7**.

**Table 2:** A comparison of photocatalytic metal nanomaterials for antimicrobial applications

Material	Shape	Size	Concentration	Irradiation	Micro‐organisms	Antimicrobial efficacy^a)^	Log reduction	Treatment parameters	Refs.
Bacteria									
TiO_2_	Not specified	79 nm	100 ppm	310 ≤ λ ≤ 400 nm	*E. coli*	75%	NR	13.6 W m^−2^, 6 h	^[^ ^112^ ^]^
Ni‐doped TiO_2_	Sphere	8–10 nm	1 mg mL^−1^ Ni‐TiO_2_ in 5 mL saline water	λ > 400 nm	*S. aureus* *E. coli* *Salmonella albony* *B. subtilis*	>99% (4 h) >99% (5 h) >99% (6 h) >99% (4 h)	4 3.5 3.3 4.3	≈0.5 mW cm^−2^, 0–6 h treatment	^[^ ^183^ ^]^
Cu‐doped TiO_2_	Sphere	8–12 nm	1 mg mL^−1^ Cu‐TiO_2_ in 5 mL saline water	λ > 400 nm	*S. aureus* *E. coli*	>99% (2 h) >99% (4 h)	NR NR	≈0.5 mW cm^−2^, 0–4 h treatment	^[^ ^184^ ^]^
Carbon‐doped TiO_2_	Not specified	Not specified	1 g L^−1^	λ > 385 nm λ > 455 nm	*E. coli*	>99% ≈80%	NR NR	1.8 W cm^−2^ (385 nm) 1.0 W cm^−2^ (455 nm), 2 h	^[^ ^204^ ^]^
TiO_2_‐graphene	Sphere	37 nm	995 mg mL^−1^	λ > 400 nm	*E. coli*	64%	0.443	450 W xenon lamp, ≈18 cm above sample, 440 min	^[^ ^185^ ^]^
S‐doped TiO_2_	Sphere	10 nm	0.2 mg mL^−1^	λ > 420 nm	*Micrococcus lylae*	>95%	NR	≈47 mW cm^−2^, 1 h	^[^ ^188^ ^]^
Ag–TiO_2_	Composites	12.7–22.8 nm	200 × 10^−9^ m	Visible light (not specified)	*E. coli*	>99%	NR	Not specified 6 h	^[^ ^205^ ^]^
CdS	Spheroids	5–65 nm	0.1 µg mL^−1^ 0.5 µg mL^−1^ 1 µg mL^−1^	λ > 420 nm	*E. coli* *S. aureus*	≈60 ≈80 >95% ≈55% ≈80% >90%	NR	300 W Xe lamp (does not specify distance from sample), 4 h	^[^ ^189^ ^]^
ZnO	Not specified	Not specified	0.01 mg mL^−1^	250 ≤ λ ≤ 400 nm	*E. coli* *P. aeruginosa* *S. aureus*	>99% (40 min) >99% (40 min) >99% (2 h)	5 5 5	400 W sodium lamp ≈10 cm above sample, 0–4 h	^[^ ^195^ ^]^
ZnO	Rods	186 nm length 20 nm width	1 g L^−1^	365 ≤ λ ≤ 750 nm	*E. coli*	≈20%	0.07	5.5 mW cm^−2^, 3 h	^[^ ^193^ ^]^
n‐HA/ZnO	Rod	80–90 nm length 15–30 nm diameter	1 g mL^−1^ 1 g mL^−1^	UV light (not specified)	*S. aureus* *E. coli*	99.45% 95.65%	NR NR	Not specified	^[^ ^196^ ^]^
nFe_2_O_4_–Ag–rGO	Particles and clusters bound to sheets	Not specified	250 mg L^−1^	λ > 400 nm	*E. coli*	>99%	7.2	300 W Xe lamp (does not specify distance from sample), 90 min	^[^ ^197^ ^]^
Ag_3_PO_4_ GO‐AG_3_PO_4_	Rhombic Dodecahedral	500 nm	20 mg L^−1^	420 ≤ λ ≤ 630 nm	*E. coli*	>99% >99%	NR NR	80 mW cm^−2^, 2 h	^[^ ^198^ ^]^
Au Ni Si Ag	Spheres	20–30 nm	10 mg L^−1^ 10 mg L^−1^ 10 mg L^−1^ 50 µg L^−1^	λ = 356 nm	*E. coli*	≈10% ≈60% ≈80% ≈95%	Check again	0.78 mW cm^−2^, 2 h	^[^ ^114^ ^]^
BiVO_4_	Octahedral	200–300 nm	50 mg L^−1^	Visible light (not specified)	*E. coli*	88%	NR	100 mW cm^−2^, 1 h	^[^ ^192^ ^]^
CuO	Rods	2.2 ± 0.67 µm length 70.1 ± 14.7 nm width	5 ppm 10 ppm 20 ppm	λ > 400 nm	*E. coli*	pH 6.0 ≈40% ≈70% ≈92% pH 7.0 ≈30% ≈35% ≈45%	NR	pH 6.0 pH 7.0 15.6 mW cm^−2^, 2 h	^[^ ^199^ ^]^
Fe‐doped CuO	Spheres	21 nm	100 µg mL^−1^	Not specified	*S. aureus* *S. epidermidis*	≈20% ≈20%	NR	Not specified, 24 h	^[^ ^201^ ^]^
TiO_2_	Not specified	Not specified	10 mg mL^−1^	315 ≤ λ ≤ 400	*E. coli* *P. aeruginosa* *C. freundii* *S. aureus* *S. sapprophyticus*	>99% >99% >99% >99% >99%	NR	11 W, 20 min	^[^ ^200^ ^]^
ZnO	Not specified	Not specified	10 mg mL^−1^	315 ≤ λ ≤ 400	*E. coli* *P. aeruginosa* *C. freundii* *S. aureus* *S. sapprophyticus*	>99% ≈97% ≈92% ≈90% ≈37%	NR	11 W, 20 min	^[^ ^200^ ^]^
Fluorinated‐SnO_2_	Hollow spheres	100–200 nm (Cavity ≈50 nm)	500 mg L^−1^	λ > 365 nm	*E. coli*	>99%	7.5	15 W, 150 min	^[^ ^191^ ^]^
Ag–chitosan–TiO_2_	Composites	≈50 nm	3.0 mg mL^−1^	UV–vis (central wavelength 365 nm)	*E. coli* *S. aureus*	>99% >99%	6 3.5	20 W, 2 h	^[^ ^206^ ^]^
Fungi									
Ag–chitosan–TiO_2_	Composites	≈50 nm	3.0 mg mL^−1^	UV–vis (central wavelength 365 nm)	*C. albicans*	>99%	4	20 W, 2 h	^[^ ^206^ ^]^
TiO_2_	Not specified	Not specified	10 mg mL^−1^	315 ≤ λ ≤ 400	*Aspergillus fumigatus* *Penicillum spp*.	>99% >99%	NR	11 W, 3 h	^[^ ^200^ ^]^
ZnO	Not specified	Not specified	10 mg mL^−1^	315 ≤ λ ≤ 400	*Aspergillus fumigatus* *Penicillum spp*.	>99% >99%	NR	11 W, 3 h	^[^ ^200^ ^]^
Carbon‐doped TiO_2_	Not specified	Not specified	1 g L^−1^	λ >385 nm λ > 455 nm	*C. albicans*	≈80% ≈20%	NR	1.8 W cm^−2^ (385 nm) 1.0 W cm^−2^ (455 nm), 2 h	^[^ ^204^ ^]^
PdO‐modified N‐doped TiO_2_	Nonuniform	10–20 nm	1 mg mL^−1^	λ > 400 nm	*Fusarium graminearum*	>99%	3.5	20 mW cm^−2^, 5 h	^[^ ^207^ ^]^
TiO_2_	Spheres	30 nm	0.1 g L^−1^	λ > 340 nm	*Saccharomyces cerevisiae* *Botrytis cinerea* *Candida krusei* *Rhodotorula glutinis*	>99% <1% >99% ≈90%	7 0 6.8 1	3.8 mW cm^−2^, 5 h	^[^ ^208^ ^]^
Fe‐doped CuO	Spheres	21 nm	100 µg mL^−1^	Not specified	*C. albicans*	≈85%	NR	Not specified, 24 h	^[^ ^201^ ^]^
Au–methylene blue	Spheroids	21 ± 2.5 nm	20 µg mL^−1^	λ = 660 nm	*C. albicans*	82.2%	NR	120 mW, 40 s	^[^ ^202^ ^]^

a) Antimicrobial efficacy may be due to combinatorial effects with other antimicrobial mechanisms in some cases. NR: Not reported.

**Figure 8:**
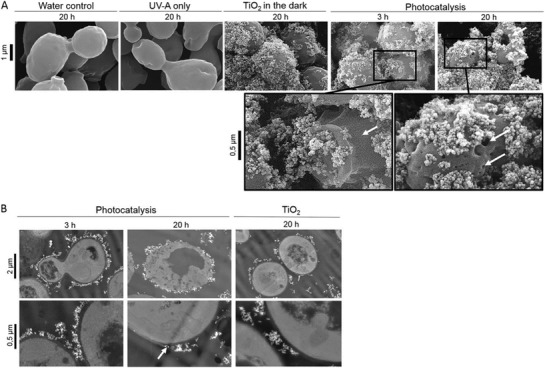
Fungal cell membrane damage as a result of photocatalytic TiO_2_ nanoparticles. A) Scanning electron micrographs of *S. cerevisiae* cells exposed to controls (20 h in water, UV‐A, or TiO_2_ in the dark) or treatment (3 and 20 h) under light irradiation with photocatalytic nanoparticles. B) Transmission electron micrographs of *S. cerevisiae* cells after 3 and 20 h of photocatalytic treatment as well as after 20 h of exposure to nonilluminated TiO_2_. White arrows indicate cell wall cracks and holes. Reproduced with permission.^[^ ^208^ ^]^ Copyright 2014, American Society of Microbiology.

Research into the use of photocatalytic metal nanomaterials against fungi has not been as extensive as that performed for bacteria and while some studies have shown some very promising results, the differences between fungal species have been shown to significantly influence the extent of antimicrobial efficacy. Early studies assessing the antifungal potential of photocatalytic metal nanomaterials were focused on the important fungal species involved in the process of food spoilage.^[^ ^209^ ^]^ In the context of medically relevant, pathogenic fungi, a milestone study was performed by Mitoraj et al. who doped TiO_2_ with carbon and tested the antimicrobial activity against the human pathogen *C. albicans* under irradiation with UV–vis (>385 nm) and visible (>455 nm) light. These authors found contrasting responses, with only ≈20% inactivation under visible light, while this rose to ≈80% under UV–vis irradiation.^[^ ^204^ ^]^ In a more comprehensive study, Thabet et al. measured the photocatalytic effect on a range of fungi, incubating *Saccharomyces cerevisiae*, *Botrytis cinerea*, *Candida krusei*, and *Rhodotorula glutinis* with commercially purchased TiO_2_ nanospheres for 20 h, taking measurements every 5 h and calculating the viable colony forming units of the solution through dilution series and plate counts.^[^ ^208^ ^]^ After 5 h, they demonstrated >99% inactivation of *S. cerevisiae* and *C. krusei* and ≈90% inactivation of *R. glutinis*, which rose to >99% following a 20 h incubation; the antifungal activity can be visualized by electron microscopy, as shown in **Figure** **8**. Contrastingly, *B. cinerea* appeared relatively unaffected by this light exposure, which the authors suggested was due to a combination of factors such as: the presence of a thick cell wall consisting of polysaccharides, the protective role of melanins and carotenoid pigment, the inability for nanoparticles to adsorb to the fungal cell wall (unexplained) and the accumulation of polyols, which may serve an antioxidative function (e.g., mannitol).^[^ ^208^ ^]^ Alternate materials to TiO_2_ have also been investigated for their photocatalytic antifungal efficacy such as ZnO, which has been shown to almost completely inactivate *Aspergillus fumigatus* and *Penicillum spp*.^[^ ^200^ ^]^ as well as Fe‐doped CuO, which was used against *C. albicans* with an observed >99% decrease in cell viability.^[^ ^201^ ^]^ A summary of studies observing the antifungal activity of photocatalytic metal nanomaterials can be found in Table 2. While the use of photocatalytic metal nanomaterials against fungi has been shown to be promising, natural resistance mechanisms possessed by fungi may limit the efficacy of this treatment against a broad spectrum of pathogenic fungal species. To this end, future antifungal photocatalytic nanomaterial designs should focus on developing broad‐spectrum antifungal agents, either through increasing the efficacy of the photocatalytic effect, or more rationally, combining this activity with a secondary antifungal mechanism to enable synergistic effects.

#### 6.1.3 Bioconjugation for Targeted Activity

**Figure 9:**
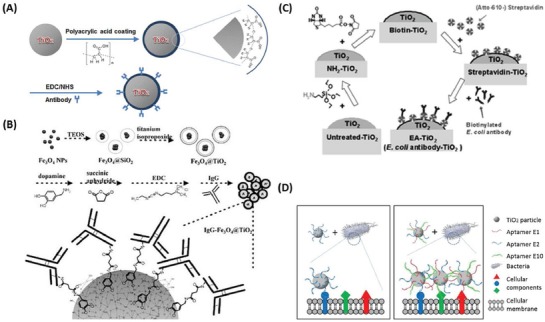
Schematic representations of the synthesis methods for bioconjugating antibodies to antimicrobial metal nanomaterials and aptamer–cell interactions. A) Schematic illustration of the preparation of bacterial target‐specific TiO_2_ particles, where TiO_2_ particles are surface‐coated with polyacrylic acid (PAA), followed by conjugation of a polyclonal antibody via an EDC/NHS coupling reaction. Reproduced with permission.^[^ ^194^ ^]^ Copyright 2014, Elsevier. B) Preparation steps for fabricating IgG‐Fe_3_O_4_@TiO_2_ magnetic nanoparticles. A thin layer of silicate is first immobilized on the bare iron oxide nanoparticles followed by coating with another layer of titania. The particles are then suspended in a dopamine solution, allowing dopamine molecules to attach. The dopamine‐immobilized Fe_3_O_4_@TiO_2_ nanoparticles are reacted with succinic anhydride. After carboxylate terminals are generated on the surfaces of the magnetic nanoparticles, IgG molecules can be readily bound to the nanoparticles through amide bonding. Reproduced with permission.^[^ ^212^ ^]^ Copyright 2008, Wiley‐VCH. C) Preparation of TiO_2_ nanoparticles with *E. coli* antibodies, through streptavidin–biotin interactions. Reproduced with permission.^[^ ^211^ ^]^ Copyright 2013, ACS Publications. D) Schematic representation of TiO_2_ particles conjugated with a single aptamer (left) or an aptamer cocktail (right), and their binding difference on the cellular surface. Reproduced with permission.^[^ ^213^ ^]^ Copyright 2016, Elsevier.

An important criterion for new antimicrobial treatment methods is their capacity to selectively target the pathogenic micro‐organism of interest. The rationale for this design is to reduce cytotoxic side effects, while simultaneously decreasing the widespread development of antimicrobial resistance; as was the case for traditional antibiotics. Additionally, following the recent paradigm shift in thinking about health as a combination of the human body and the microbiome that inhabits it, off‐targeted antimicrobial effects can often have more negative outcomes than the primary infection.^[^ ^210^ ^]^ The main strategy for developing targeted metal nanomaterials is through bioconjugation of molecules, such as antibodies or aptamers, which selectively bind to a particular pathogenic species or strain of interest. For example, Ye et al. demonstrated a decrease of viable *E. coli* on the order of 10^4^ cells from the control group after 120 min treatment using TiO_2_ nanoparticles bound to *E. coli* antibodies.^[^ ^211^ ^]^ TiO_2_ nanoparticles were first treated with 3‐aminopropyltriethoxysilane, followed by *N*‐hydroxysuccinimidobiotin to create biotinylated TiO_2_ nanoparticles, which were mixed with a streptavidin solution. Biotinylated *E. coli* antibody solution was added, which bound to the streptavidin TiO_2_ nanoparticles through a sequential process of addition, ultrasonication and centrifugation (**Figure** **9**C). They found that the *E. coli* antibody‐bound TiO_2_ nanoparticles caused flocculation of the bacterial cells and a significant reduction in concentration of *E. coli* following irradiation with light in the UV range, assessed through coliform specific plate counts. Importantly, they found no significant reduction in the concentration of *Pseudomonas putida*, which was added to the solution as a representative “non‐targeted species.”^[^ ^211^ ^]^ In addition to the advantage of selective targeting of bioconjugated nanoparticles, these authors also found them to be more effective overall than nonconjugated particles. This is likely a result of the close proximity of the bound nanoparticles with the bacteria allowing a higher proportion of the generated ROS species to act on the cellular membrane, which would otherwise be lost to the surrounding medium.

Additional examples of the bioconjugation of photocatalytic nanomaterials include Song et al. who used polyacrylic acid, as opposed to streptavidin‐biotin, to bind *E. coli* antibodies to TiO_2_ nanoparticles (Figure 9A); demonstrating 90% inactivity following a 15 min treatment period.^[^ ^194^ ^]^ Interestingly, Chen et al. were able to utilize Fe_3_O_4_/TiO_2_ core/shell magnetic nanoparticles, bound with bacterial antibodies for selective antibacterial targeting (Figure 9B), while the magnetic properties allowed selective control of the movement of the particles in vivo.^[^ ^212^ ^]^ In addition to antibodies, aptamers, which are specialized oligonucleotides or peptides, can be utilized to selectively bind to the target pathogenic bacteria. For example, Song et al. demonstrated that TiO_2_ nanoparticles bound with aptamers were more effective at reducing the concentration of *E. coli* than unbound nanoparticles (Figure 9D). Interestingly, they found that TiO_2_ nanoparticles bound with multiple aptamers demonstrated increased levels of inactivation of *E. coli* cells than nanoparticles bound with single aptamers, likely due to the increased selectivity generated by the multiple different aptamers, which can simultaneously target different bacterial molecules present in the same bacteria.^[^ ^213^ ^]^ Such selectivity is important in vivo where the pathogenic species may be in a mixed culture environment.

#### 6.1.4 Challenges and Future Outlooks for Antimicrobial Photocatalytic Metal Nanomaterials

Photocatalytic nanomaterials demonstrate considerable promise as candidates for antimicrobial applications. Since the first proof of principle study investigating the use of photocatalytic nanomaterials for their antimicrobial activity in 1985, researchers have developed antimicrobial photocatalytic nanomaterials from a wide variety of materials with high antimicrobial efficacy against a broad spectrum of pathogenic micro‐organisms, as summarized in Table 2. Targeted methods of delivery have also found success through bioconjugation of the nanomaterials with pathogen‐specific antibodies and aptamers.

While the use of photocatalytic metal nanomaterials against fungi has shown promise, natural resistance mechanisms possessed by fungi may limit the efficacy of this treatment against a broad spectrum of pathogenic fungal species. Additionally, despite successful targeting of specific bacterial species in vitro through bioconjugation, the oxygen free radicals produced by these photocatalytic nanomaterials may cause damage to the mammalian cell membrane and hence the proposition of cytotoxicity is still a prominent issue.

For this technology to be clinically successful, future strategies require the incorporation of well‐thought‐out design principles and analytical approaches, before testing the antimicrobial activity. Importantly, materials and combinations of materials that possess conduction and valence band edges above and below (respectively) the required redox potentials of relevant ROS reactions should be explored (illustrated in Figure 5). Concurrently, careful consideration of the total bandgap required to initiate the reaction is required. This should ideally remain within the energy levels equivalent to light in the NIR wavelengths, which are biomedically relevant. This can be simply calculated by utilizing Planck's constant (*h*)

(1) E = hc/λ

where *c* is the speed of light and λ is the wavelength of the photon. For example, the wavelengths which are thought to be able to penetrate into deep tissue (650 ≤ λ ≤ 950 and 1000 ≤ λ ≤ 1350) are equivalent to ≈1.9–1.3 and 1.2–0.9 eV, respectively. For this reason, it is not possible to develop photocatalytic nanomaterials that can drive all the ROS reactions described in Table 1, as the total bandgap required would be too large. A simple strategy would be to utilize separate nanomaterials that have similarly small bandgaps, but different conduction and valence band edges, which can cover the entire range of ROS redox reactions. Furthermore, the development of Janus nanoparticles, composed of two or more semiconducting materials, may enable such activity to occur within a single particle, with enhanced photocatalytic activity.^[^ ^214^ ^]^ Thorough assessments of bioconjugated nanomaterials to improve targeting of specific bacteria and fungi are necessary, as well as comprehensive in vivo experiments to determine the cytotoxic, or other potential, side effects of photocatalytic nanomaterials.

### 6.2 Photothermal Antimicrobial Metal Nanomaterials

#### 6.2.1 Antimicrobial Mechanism of Photothermal Metal Nanomaterials

In addition to photocatalysis, the absorbance of light by metal nanomaterials can be utilized to induce rapid and considerable localized temperature increases through photothermal effects. Photothermal therapy was traditionally developed as a targeted treatment method for tumor cells, which used specific light‐absorbing dyes;^[^ ^215^ ^]^ however, recent advances in nanotechnology have allowed the development of nanomaterials that can convert light to heat.^[^ ^216^ ^]^ Photothermal activity generated from nanomaterials is highly efficient and the photothermal activity can be readily tuned to specific wavelengths by altering their size and/or shape.^[^ ^71^, ^217^ ^]^ As such, photothermal nanomaterials have been proposed as a promising solution as a targeted treatment of pathogenic micro‐organisms as they are controllable and can be localized to the immediate area surrounding the nanomaterial.^[^ ^71^, ^166^, ^218^, ^219^ ^]^

**Figure 10:**
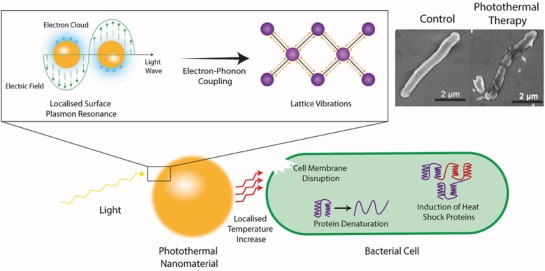
Schematic representation of the photothermal conversion of light to heat and the subsequent antimicrobial mechanism taking place. The electromagnetic field causes electrons in the conduction band at the surface of the metal nanomaterials to rapidly oscillate. This absorbed energy induces vibrations in the metallic lattice, via electron–phonon coupling, which is subsequently transferred into thermal energy resulting in a localized increase in temperature which is responsible for the antimicrobial activity. Top right are scanning electron micrographs of *E. coli* cells before (left) and after (right) treatment with photothermal nanomaterials. Reproduced with permission.^[^ ^232^ ^]^ Copyright 2018, Wiley‐VCH.

Nanomaterials composed of certain metals possess the phenomena of localized surface plasmon resonance (LSPR) when exposed to light at specific wavelengths. In short, at a wavelength close to the size of the metal nanomaterial, the electromagnetic field causes electrons in the conduction band at the surface of the metal nanomaterials to oscillate, creating a rapidly moving electron cloud.^[^ ^71^, ^220^, ^221^ ^]^ This absorbed energy can be dissipated either by re‐emitting a photon, or via heat through electron–electron interactions and then electron–phonon relaxation, which induces vibrations in the metal lattice structures, these lattice vibrations are transferred into thermal energy causing localized heat around the nanomaterial^[^ ^44^, ^71^, ^220^ ^]^ (**Figure** **10**). This phenomenon has been predominately studied using gold; however silver,^[^ ^222^ ^]^ copper,^[^ ^223^ ^]^ and other materials^[^ ^224^ ^]^ have also been investigated. By conjugating specific attachments to the nanomaterials, such as antibodies, they can specifically target the pathogen of interest, where the localized increase in temperature causes cell death through a suite of actions including denaturation of essential proteins/enzymes, induction of heat shock proteins, disruption of metabolic signaling and rupture of the cell membrane^[^ ^71^, ^85^, ^218^, ^219^, ^225^, ^226^, ^227^ ^]^ (Figure 10).

LSPR is determined by the electron charge density at the surface of the nanomaterial, which is affected by the size, shape, and composition of the nanomaterial and hence can be altered and controlled.^[^ ^228^ ^]^ For example, nonspherical nanomaterials can possess multiple excitation peaks as the shape allows different ways in which the electron clouds can be polarized. Furthermore, increased edges and/or aspect ratio of the nanomaterial typically results in a shift toward longer excitation wavelengths due to charge separation, while increasing the size of the nanomaterial also increases the excitation wavelength.^[^ ^229^ ^]^ Additionally, the composition can also shift the excitation wavelength, for example, when assessing the LSPR of similar sized nanocubes on identical substrates, it was found that gold excites at longer wavelengths.^[^ ^230^ ^]^ Advances in nanomaterial synthesis have allowed the design of customized nanomaterials such as gold nanorods,^[^ ^231^ ^]^ nanoshells,^[^ ^80^ ^]^ nanocages,^[^ ^81^ ^]^ nanostars,^[^ ^82^ ^]^ nanopopcorn,^[^ ^85^ ^]^ and a variety of other unique shapes and similarly unique names.^[^ ^219^ ^]^ The advantage of these shapes is that the specific SPR wavelength can be shifted into the biological NIR windows, which largely passes through human cells and tissues making in vivo applications of this technology very promising.

#### 6.2.2 Antimicrobial Activity of Photothermal Metal Nanomaterials

**Figure 11:**
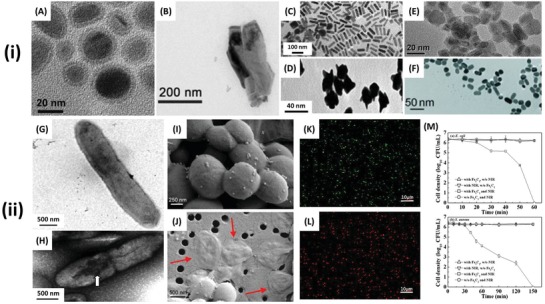
i) TEM images of antibacterial, photothermal nanomaterials. A) Fe_5_C_2_ spheroids. Reproduced with permission.^[^ ^235^ ^]^ Copyright 2015, Royal Society of Chemistry. B) In_2_Se_3_ nanosheets. Reproduced with permission.^[^ ^232^ ^]^ Copyright 2018, Wiley‐VCH. C) Gold nanorods. Reproduced with permission.^[^ ^226^ ^]^ Copyright 2008, ACS Publications. D) Monoclonal M3038 antibody‐conjugated popcorn shaped gold nanoparticles. Reproduced with permission.^[^ ^85^ ^]^ Copyright 2011, Royal Society of Chemistry. E) BSA–CuS platelike particles. Reproduced with permission.^[^ ^223^ ^]^ Copyright 2017, ACS Publications. F) Anti‐salmonella‐antibody‐conjugated oval‐shaped gold nanoparticles. Reproduced with permission.^[^ ^234^ ^]^ Copyright 2010, Wiley‐VCH. ii) Visualization and quantification of the antibacterial activity of photothermal nanomaterials. TEM images of *P. aeruginosa* bound by antibody conjugated nanorods G) before and H) after 10 min NIR irradiation. The white arrow indicates an area of the bacterial cell surface that has suffered irreparable damage. Reproduced with permission.^[^ ^226^ ^]^ Copyright 2008, ACS Publications. SEM images showing *S. aureus* treated with functionalized gold nanoparticles I) control and J) exposed to pulsed laser irradiation at 532 nm. Red arrows indicate damaged bacterial cells. Reproduced with permission.^[^ ^225^ ^]^ Copyright 2015, Dove Press. Confocal laser scanning microscopy (CLSM) images of *E. coli* exposed to BSA–CuS nanoparticles K) before and L) after NIR irradiation. Bacterial viability determined through live/dead staining where green indicates live bacteria and red indicates bacteria with significant membrane damage. Reproduced with permission.^[^ ^223^ ^]^ Copyright 2017, ACS Publications. M) Inactivation effects of Fe_5_C_2_ nanoparticles (50 mg L^−1^, pH 6) and NIR irradiation for *E. coli* (top) and *S. aureus* (bottom). Error bars represent standard deviations from triplicate experiments (*n* = 3) . Reproduced with permission.^[^ ^235^ ^]^ Copyright 2015, Royal Society of Chemistry.

A landmark study for the use of antimicrobial photothermal nanomaterials was compiled by Zharov et al. using antibody conjugated, spherical gold nanoparticles with diameters ≈10, 20, and 40 nm, against *S. aureus*. They observed significant decreases in their viability following the addition of the 40 nm conjugated gold nanoparticles and laser irradiation at 532 nm, when compared to control samples which were exposed to only either the nanoparticles or the laser. A dual function bactericidal mechanism was proposed, in which the bacterial cell wall is disrupted through a combination of localized heating and bubble formation by the gold nanoparticles attached to the bacterial cell wall.^[^ ^233^ ^]^ Since then, there have been numerous studies investigating the antimicrobial activity of photothermal metal nanomaterials such as Huang et al. who were able to successfully attach vancomycin, which binds to specific peptides on the cell wall, to polyglonal gold nanoparticles which demonstrated the prospects of targeted antibacterial activity of photothermal nanomaterials.^[^ ^218^ ^]^ Conjugation of antibodies is also possible, for example, Norman et al. were able to covalently bind *P. aeruginosa* antibodies to gold nanorods and selectively target and inactivate ≈75% of *P. aeruginosa* cells following exposure to NIR radiation (λ = 785 nm) for only 5 min;^[^ ^226^ ^]^ among others.^[^ ^225^ ^]^ Similarly, Wang et al. were able to bind anti‐salmonella antibodies onto oval‐shaped gold nanoparticles, where they found ≈90% inactivation following a 10 min exposure to 785 nm laser irradiation.^[^ ^234^ ^]^ To bind the antibodies onto the gold nanoparticles, first they capped the nanoparticle in a bilayer of positively charged CTAB. Following this, the nanoparticle surface was modified with amine groups using cystamine dihydrochloride and the antibody was covalently bound using a glutaraldehyde spacer method.^[^ ^234^ ^]^ The successful bioconjugation of photothermal nanomaterials shows promise toward the selectivity of this treatment method. The range of metal nanomaterials used for antibacterial photothermal activity can be visualized in **Figure** **11**.

While gold is the most commonly used material for photothermal therapy, researchers have also found success with other metal nanomaterials, highlighting the versatility of this treatment method. For example, Huang et al. used copper–sulfide (CuS) nanocomposites, bioconjugated with bovine serum albumin (BSA) to improve the biocompatibility of the nanocomposites. They demonstrated over 80% cell death of both the Gram‐positive *S. aureus* and Gram‐negative *E. coli* following the addition of 50 ppm of the BSA–CuS nanocomposites and irradiation to a NIR laser at 980 nm for 45 min.^[^ ^223^ ^]^ Additionally, D'Agostino et al. utilized triangular silver nanoplates, with sides approximately 200 nm, which demonstrated inactivation of *S. aureus* (97%) and *E. coli* (>99%) cells respectively, following irradiation at 808 nm (260 mW cm^−2^), for 15 min.^[^ ^222^ ^]^ Interestingly, they were able to affix the nanoplates to glass surfaces to demonstrate the effectiveness of photothermal nanomaterials as a stimuli‐activated surface coating, for purposes such as biomedical implants.^[^ ^222^ ^]^ Furthermore, they explored the relationship between the nanoscale dimensions of the triangular nanoplates and the specific excitation wavelength to initiate the photothermal response, highlighting the controllability of this technique, which will logically become easier to manipulate as synthesis processes of metal nanomaterials continue to rapidly improve. Additional materials have also been utilized, such as vancomycin‐modified polyelectrolyte‐cypate coated silica nanoparticles (SiO_2_‐Cy‐Van), which were found to possess successful activity against methicillin‐resistant *S. aureus* both in vitro and in vivo following 5 min irradiation at 808 nm.^[^ ^224^ ^]^ Further studies and materials used for antimicrobial photothermal nanomaterials can be found in **Table** **3**.

**Table 3:** A comparison of photothermal metal nanomaterials for antimicrobial applications

Material	Shape	Size	Concentration	Irradiation	Micro‐organisms	Antimicrobial efficacy^a)^	Log reduction	Treatment parameters	Refs.
Bacteria									
Au	Sphere	10 nm 20 nm 40 nm	NR	λ = 525 nm	*S. aureus*	≈20% ≈60% ≈90%	NR	100 laser pulses. Pulse width, 8 ns; and pulse energy, 1 µJ (0.2 J cm^−2^)	^[^ ^233^ ^]^
Au	Star	50–100 nm	1.1–1.4 × 10^14^ cm^−2^ (surface coverage)	λ = 808 nm	*S. aureus*	≈96%	1.5	90 mW cm^−2^, 30 min	^[^ ^82^ ^]^
Au	Rod	18 nm width 68 nm length	NR	λ = 785 nm	*P. aeruginosa*	75%	NR	≈50 mW, 10 min	^[^ ^226^ ^]^
Au	Oval	≈10–30 nm width ≈20–40 nm length	NR	λ = 670 nm	*Salmonella typhimurium*	90%	NR	40 mW, 10 min	^[^ ^234^ ^]^
Au	Popcorn	Not specified (≈15–40 nm)	NR	λ = 670 nm	*Salmonella typhimurium*	>99%	NR	200 mW cm^−2^, 20 min	^[^ ^85^ ^]^
Van‐Fe_3_O_4_–Au	Eggs	50–100 nm	168.75 µg mL^−1^	λ = 808 nm	*Acinetobacter baumannii* *E. coli* *Staphylococcus pyogenes*, *Staphylococcus saprophyticus* *Enterococcus faecalis* *Enterococcus faecium*	>99% >99% >99% >99% >99% >99%	NR	250 mW cm^−2^, 3 min	^[^ ^84^ ^]^
CuS	Plate‐like	12–28 nm diameter	50 ppm (Cu^2+^ ions)	λ = 980 nm	*S. aureus* *E. coli*	>80% >80%	NR	1.59 W cm^−2^, 45 min	^[^ ^223^ ^]^
Ag	Triangular plates	≈200 nm side length	1.87 × 10^−6^ g cm^−2^	λ = 808 nm	*S. aureus* *E. coli*	97% > 99%	1.5 2.5	260 mW cm^−2^, 15 min	^[^ ^222^ ^]^
SiO_2_‐Cy‐Van	Sphere	72.7 ± 3.2 nm	81.82 mg (loaded with 150 µg Cy and 2.05 µg Van) injected into mouse	λ = 808 nm	*S. aureus*	Qualitatively significant cell death (no quantitative statistics reported)	NR	1.5 W cm^−2^, 5 min	^[^ ^224^ ^]^
Fe_3_O_4_–Au	Necklace (Rods surrounded by spheres)	Rods 55 nm long 20 nm wide Spheres 15 nm	NR	λ = 785 nm	*E. coli* *S. typhimurium*	Qualitatively suggests cell death (no quantitative statistics reported)	NR	50 mW, 15 min	^[^ ^236^ ^]^
Fe_3_O_4_‐alumina	Core–shell (sphere)	NR	80 µg mL ^−1^	λ = 808 nm	*Acinetobacter baumannii* *E. coli* *Enterococcus faecalis* *Streptococcus pyogenes*	≈63% (3 min) ≈83% (5 min) >99% (10 min) ≈80% (3 min) ≈90% (5 min) ≈99% (10 min) ≈47% (3 min) ≈65% (5 min) ≈97% (10 min) ≈83% (3 min) ≈93% (5 min) ≈98% (10 min)	NR	640 mW cm^−2^, 3, 5, 10 min	^[^ ^237^ ^]^
Fe_5_C_2_	Spheroid	20 nm	50 mg L^−1^	λ = 808 nm	*S. aureus* *E. coli*	>99% (150 min) >99% (60 min)	6 6	2.5 W cm^−2^, 60 min, 150 min	^[^ ^235^ ^]^
In_2_Se_3_	Sheets	300 nm	150 ppm	λ = 808 nm	*S. aureus* *E. coli*	>99% ≈98%	NR	3 W cm^−2^, 10 min	^[^ ^232^ ^]^
Fungi									
Graphene oxide	Flakes	Assorted	NR	λ = 1064 nm	*S. cerevisae* *Candida utilis*	>99% >99%	4 4	NR	^[^ ^238^ ^]^
Polymer	Sphere	60–300 nm	6 µg mL^−1^	λ = 808 nm	*C. albicans*	>99%	NR	2 W cm^−2^, 5 min	^[^ ^239^ ^]^

a) Antimicrobial efficacy may be due to combinatorial effects with other antimicrobial mechanisms in some cases.

NR: Not reported.

Studies of photothermally‐activated metal nanomaterials as antifungal agents are notably absent in the literature. A few studies have, however, investigated the use of nonmetal nanomaterials against fungal cells. For example, Khan et al. demonstrated a >99% reduction in the viability of *C. albicans* and *S. cerevisiae*, utilizing graphene oxide nanoflakes under the irradiation light in the NIR region (λ = 1064 nm).^[^ ^238^ ^]^ Furthermore, Wang and Irudayaraj demonstrated the antifungal effect of a polymer sphere when irradiated with light at wavelength 808 nm, for 5 min, observing >99% inactivation of *C. albicans* cells.^[^ ^236^ ^]^ Hence, there is an obvious need for future designs of photothermal nanomaterials to be screened against relevant fungal pathogens to determine their efficacy and underlying antifungal mechanisms.

#### 6.2.3 Challenges and the Future Outlook for Antimicrobial Photothermal Metal Nanomaterials

The use of photothermal nanomaterials for antimicrobial applications is only a recent phenomenon, first demonstrated less than two decades ago. Such materials show promise as clinical alternatives to current treatment methods and there is considerable scope for the use of photothermal nanomaterials as a stimuli‐activated, antimicrobial treatment method to supplement and/or supersede current treatment strategies. Possessing a highly tunable range of light activation into the NIR region, which can penetrate through human cells and are able to potentially be effective in deep tissue infections. However, it should be noted that even within this biological window, achievable penetration depths are only a few centimeters,^[^ ^240^ ^]^ which may not be applicable for infections in larger patients. Importantly, the antimicrobial efficacy of activated photothermal nanomaterials has been shown to occur over time periods on the order of minutes, which is clinically relevant moving forward, as opposed to photocatalytic nanomaterials whose antimicrobial activity is typically assessed over a period of hours. Furthermore, the ability to specifically target pathogenic micro‐organisms of interest through bioconjugation reduces the potential side effects of this treatment and ensures narrow spectrum efficacy, which has typically been a significant issue for antibiotics which commonly kill “good” bacteria and can shift the homeostatic microbiome paradigm of the patient in favor of “bad” bacteria, which can have serious unintended consequences.

Future research should investigate the potential of this technology against fungal pathogens, while comprehensive in vivo studies are required to determine any potential cytotoxic effects, as mammalian cells are quite susceptible to heat increases, which result in cell apoptosis and necrosis.^[^ ^241^ ^]^ Additionally, there must be a consideration in future studies to address the efficacy of a thermal‐based antimicrobial agent against infections by bacteria which are naturally resistant to high levels of heat, such as spore‐forming bacteria, which are quite common food pathogens, e.g., *Bacillus cereus* and *Chlostridium perfringens*. Overall, antimicrobial photothermal nanomaterials offer a novel solution as a stimuli‐activated antimicrobial treatment strategy, which warrants further investigation.

## 7 Magnetic Activated Antimicrobial Metal Nanomaterials

In addition to light, metal nanomaterials can be responsive to magnetic fields, which has led to the development of magnetically activated antimicrobial technologies. Magnetic activation has an innate advantage over light, whereby human tissue is largely transparent to magnetic fields, meaning that deep penetration and activation is possible. In general, two primary magnetically activated mechanisms have been explored: 1) magnetic hyperthermia, whereby magnetic activation induces localized temperatures changes, and 2) magnetophysical action, which describes the kinetically driven antimicrobial behavior of a nanomaterial in response to an applied magnetic field.

### 7.1 Magnetic Hyperthermia Antimicrobial Metal Nanomaterials

#### 7.1.1 Antimicrobial Mechanism of Magnetic Hyperthermia Metal Nanomaterials

Magnetic hyperthermia relies on the ability of magnetic nanomaterials to produce heat, in a localized manner similar to photothermal therapy.^[^ ^242^, ^243^, ^244^ ^]^ The magnetism of a particle relies on the directionality of electron spin which comprises the individual atoms. In typical ferromagnetic materials, these spin states naturally become co‐localized in separate domains and hence are referred to as multidomain.^[^ ^245^ ^]^ When a magnetizing force is applied, the directionality of these domains align until it reaches a point of magnetic saturation, whereby the material reaches a limit of magnetic flux density and becomes permanently magnetized.^[^ ^245^ ^]^ To demagnetize the material, a magnetizing force in the opposite direction must be applied, which typically must be stronger than the initial force due to energy loss, in the form of heat.^[^ ^242^, ^245^ ^]^ This phenomena is also known as hysteresis loss and can be quantified through the area of the hysteresis loop for a given system.^[^ ^242^, ^245^ ^]^ The amount of hysteresis loss is governed by two primary components, 1) remanence, which is the quantification of the remaining magnetization of the material when the external magnetic field is removed and 2) coercivity, which is the value of the field in the reverse direction required to drive the magnetization of the material back to zero.^[^ ^246^ ^]^ Ferromagnetic materials, such as iron, copper and/or nickel, have previously been used for magnetic hyperthermia treatment of tumor tissues.^[^ ^247^, ^248^ ^]^

**Figure 12:**
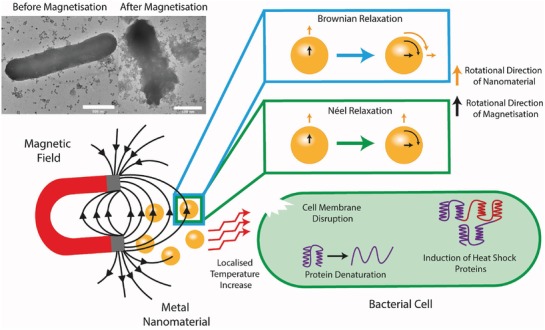
Schematic representation of the mechanism of magnetic hyperthermia of superparamagnetic nanomaterials and subsequent antimicrobial mechanism. The conversion of an external magnetic field to heat occurs due to Brownian relaxation, in which the entire nanomaterial rotates and Néel relaxation, in which only the magnetization rotates, generating localized increases in temperature which is responsible for the antimicrobial activity. Top left, scanning electron micrographs of *E. coli* before (left) and after (right) magnetic hyperthermia treatment. Reproduced with permission.^[^ ^255^ ^]^ Copyright 2017, Wiley‐VCH.

When magnetic materials drop below a critical size, it becomes energetically favorable to form only a single magnetic domain, which dramatically changes their magnetic properties.^[^ ^242^, ^243^, ^245^, ^249^, ^250^ ^]^ The critical size to form single domains is dependent on the material but typically occurs within the nanoscale range, for example estimations include 14, 55, 70, 128, and 166 nm for Fe, Ni, Co, Fe_3_O_4,_ and γ‐Fe_2_O_3_, respectively.^[^ ^251^ ^]^ Single domain nanomaterials demonstrate the unique properties of superparamagnetism, which unlike typical ferromagnetic materials do not reach a state of permanent magnetism, but rather demagnetize instantaneously when the field is removed.^[^ ^242^, ^243^ ^]^ Contrary to multidomain ferromagnetic materials, superparamagnetic nanomaterials do not display coercivity or remanence values and hence heat generated due to hysteresis loss in superparamagnetic nanomaterials is negligible.^[^ ^252^, ^253^ ^]^ Alternatively, heat is generated through relaxation losses of which there are two main types: 1) Néel relaxation, which occurs due to a delay in the relaxation time compared to the magnetic reversal time resulting in thermal energy loss and 2) Brownian motion, which describes the rotation of the nanomaterial around its axis and can become limited in highly viscous mediums or when such nanomaterials are immobilized.^[^ ^243^, ^244^, ^245^, ^253^, ^254^ ^]^ Within time‐varying magnetic fields, the relative frequency also plays a role, such that Brownian relaxation dominates in low frequency regimes, while Néel relaxation dominates at higher frequencies.^[^ ^254^ ^]^ A schematic representation of these processes and the subsequent antimicrobial effects of the magnetic hyperthermia nanomaterials is provided in **Figure** **12**.

**Figure 13:**
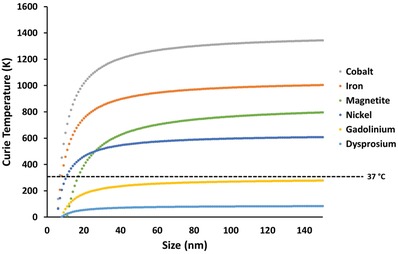
Size‐dependent Curie temperatures of various magnetic materials. Theoretical values calculated from the formulas derived from ref. [256] and represent spherical nanoparticles.

The advantages of superparamagnetic nanomaterials are a significantly higher specific absorption rate (SAR), compared to multidomain ferromagnetic materials, which is a measure of the heat‐producing capacity of such particles.^[^ ^243^ ^]^ Additionally, the temperature range can be more easily controlled, as heat production can be initiated, and subsequently halted, almost instantaneously through exposure to, and removal of, an external alternating current (AC) magnetic field. This is a very important factor in the success of these particles in vivo, to avoid unwanted cytotoxic side effects, with healthy human cells being very susceptible to high temperatures.^[^ ^241^ ^]^ Furthermore, there is an alternative strategy toward temperature control which involves synthesizing metal alloys which possess a Curie temperature near the border where thermoablation of human cells takes place (≈>46 °C).^[^ ^244^ ^]^ The Curie temperature represents the temperature above which ferromagnetic materials lose their permanent magnetic properties, reducing the magnetic hyperthermia properties and hence self‐regulating the temperature.^[^ ^244^ ^]^ The Curie temperature is determined by factors such as material choice and size. The Curie temperature of metal nanomaterials can be modeled via Equation 2 ^[^ ^256^ ^]^

(2) $$T_{\text{Cn}}=\;T_{\text{Cb}}\left(1\;-\;\frac{6\mu }{n^{1/3}C^{2/3}\pi \;k^{2}}\right)$$

where *T*_Cn_ is the Curie temperature of the nanomaterial, *T*_Cb_ is the Curie temperature for the bulk material, μ is the shape factor, *n* is the atomic number of nanocrystals, *C* is the atomic number of the structure cell, and *k* is the ratio between the equivalent atomic radius and lattice parameter. For example, Equation 3 demonstrates that in the case of spherical nanoparticles, the atomic number n can be expressed as

(3) $$n\;=\;\frac{\rho^{4/3}\pi {\left(\frac{D}{2}\right)}^{\;3}}{M}\;N_{\text{A}}$$

where ρ is the density of the material, while *D* refers to the size of the nanoparticle, *M* is the molar mass, and *N*_A_ is the Avogadro constant. The above equations were used to generate the theoretical size‐dependent Curie temperatures of spherical nanoparticles for a range of magnetic materials presented in **Figure** **13**. It should be noted, however, that this phenomena does not apply to superparamagentic nanomaterials that do not display permanent magnetism or rely on hysteresis for hyperthermia.

#### 7.1.2 Antimicrobial Activity of Magnetic Hyperthermia Metal Nanomaterials

While the use of nanomaterials for magnetic hyperthermia treatment of tumor cells has been researched for a significant period of time,^[^ ^243^, ^244^, ^247^, ^249^, ^257^ ^]^ the use of this stimuli‐activated therapy as a treatment method for inactivating pathogenic microorganisms has only been investigated within the last decade.^[^ ^255^, ^258^, ^259^, ^260^, ^261^, ^262^, ^263^ ^]^ In an early, influential study Thomas et al. observed a substantial reduction (10^7^‐fold) in viable *S. aureus* cells following the addition of superparamagnetic iron oxide nanoparticles, functionalized with the organic ligands tiopronin, oxamic acid, and succinic acid, which were then exposed to an AC magnetic field (12 kA m^−1^, 1.05 MHz) with varying pulsed treatment times. Interestingly, it was demonstrated that these particles were still effective when tested the following week, however, the antibacterial efficacy was thought to decrease after a period of one month or following significant sonication, due to increased oxidation of the nanoparticles.^[^ ^258^ ^]^ Furthermore, Park et al. were able to demonstrate antibiofilm properties of superparamagnetic iron oxide nanoparticles, achieving more than a four log reduction in viable *P. aeruginosa* cells, which comprised a thick biofilm, following an 8 min exposure to a magnetic field (3 kA m^−1^, 493 kHz).^[^ ^259^ ^]^ Interestingly, Singh et al. synthesized Fe_3_O_4_–ZnO nanocomposites, which exhibited antibacterial activity against *S. aureus* and *E. coli* under the application of an external magnetic field and was also thought to have a dual antibacterial action through the production of ROS, however the latter was not comprehensively evaluated in the study.^[^ ^261^ ^]^

Extending on this work, other studies have been able to successfully conjugate antibodies onto magnetic nanomaterials for specific pathogen targeted treatment. For example, Kim et al. conjugated iron oxide nanoparticles with anti‐protein A antibody, through biotin–streptavidin binding, for the targeted treatment of *S. aureus* biofilms. They demonstrated positive results in vitro, following 3 min treatments with an alternating magnetic field (AMF) of 18, 31 and 40 kA m^−1^ with a killing efficiency at and above 99% for 31 and 40 kA m^−1^, respectively, based on bioluminescence imaging, while 18 kA m^−1^ was insufficient to disrupt the biofilm. Importantly, they also demonstrated a significant reduction of the *S. aureus* in vivo, using a mouse model, where the bacterial culture was injected just under the skin at a sub‐lethal dose. Applying a 31 kA m^−1^ field for 3 min, they found an 80% reduction in the *S. aureus* biofilm, which was significantly higher than nanoparticles bound with the nonspecific anti‐IgG antibody or no antibodies at all, with successful wound closure and no obvious adverse effects to the mice following treatment.^[^ ^260^ ^]^ Interestingly, the measured temperature on the skin at the wound margin only rose to 43 °C, which is below the temperature healthy cells are thought to begin the process of necrosis.^[^ ^241^ ^]^ Additionally, Raval et al. synthesized GM3 conjugated Fe_3_O_4_ nanoparticles, which selectively bound to the enterotoxigenic *E. coli* strain *E. coli* K99 and found a significant reduction in viability following a 2 h treatment of an external magnetic field. Importantly, they tested their conjugated magnetic nanoparticles in a mixed culture consisting of *E. coli* K99 and *E. coli* O157, where they found no significant reduction in the *E. coli* O157.^[^ ^255^ ^]^ This validates the selectivity of antibacterial magnetic nanomaterials between different strains of the same species of bacteria, an important attribute for this technology to be clinically relevant in the future. A summary of antimicrobial studies involving the use of magnetic hyperthermia metal nanomaterials is presented in **Table** **4**.

**Table 4:** A comparison of magnetic hyperthermia metal nanomaterials for antimicrobial applications

Material	Shape	Size	Concentration	Magnetic field strength	Micro‐organisms	Antimicrobial efficacy^a)^	Log reduction	Treatment parameters	Refs.
Bacteria									
Fe_2_O_3_– Fe_3_O_4_	Sphere	≈9 nm	50 mg mL^−1^	12 kA m^−1^	*S. aureus*	>99%	7	1.05 MHz, 2 min	^[^ ^258^ ^]^
Fe_3_O_4_	Sphere	Not specified	60 mg mL^−1^	3 kA m^−1^	*P. aeruginosa*	>99%	≈4.3	492 kHz, 8 min	^[^ ^259^ ^]^
Fe_3_O_4_	Sphere	100 nm	NR	18 kA m^−1^ 31 kA m^−1^ 40 kA m^−1^	*S. aureus*	No significant difference from control >99% >99%	0 ≈2 ≈3	2.1 MHz, 3 min	^[^ ^260^ ^]^
Fe_3_O_4_ Fe_3_O_4_–ZnO	Porous nanocomposite spheres	200–800 nm	2 mg mL^−1^ 2 mg mL^−1^	425 Oe (≈34 kA m^−1^)	*E. coli*	94.3% >99%	1.24 2.58	250 KHz, 1 h	^[^ ^261^ ^]^
GM3–Fe_3_O_4_	Sphere multianchored with glycoconjugate GM3	23.7 ± 1.55 nm	650 µg mL^−1^	31 kA m^−1^	*E. coli* K99	95%	NR	207 KHz, 2 h	^[^ ^255^ ^]^
PAMAM–Fe_3_O_4_	Sphere	40 nm	5 mg mL^−1^	Not specified	*E. coli*	>99%	NR	250 kHz, 10 min	^[^ ^262^ ^]^
Fungi									
Fe_3_O_4_	Sphere	8.9 nm	2.5 mg mL^−1^	10 kA m^−1^	*C. albicans*	≈80% 40 min ≈90% 60 min	NR	531.1 kHz, 40 and 60 min	^[^ ^263^ ^]^

a) Antimicrobial efficacy may be due to combinatorial effects with other antimicrobial mechanisms in some cases. NR: Not reported.

As is the case with the research surrounding photothermal nanomaterials, there is a distinct lack of studies investigating the antifungal activity of magnetic hyperthermia nanomaterials. From our literature search, we were only able to find one study which investigated this interaction. In a recent study, Chudzik et al. utilized Fe_3_O_4_ spheres, approximately 9 nm in diameter, which would be assumed as a single domain and hence considered to be superparamagnetic. Testing against *C. albicans* fungi, they demonstrated an 80% and 90% reduction in cell viability following a 40 and 60 min magnetic (10 kA m^−1^, 531.1 kHz) treatment, respectively.^[^ ^263^ ^]^ Clearly, there is a large scope for further research in this area, however more systematic studies are required before a comprehensive evaluation of the use of this technology against fungal pathogens can be made. Future studies need to test a range of fungal species, alter the size/shape/composition of the magnetic particles, test using different magnetic parameters, investigate the use of bioconjugated nanomaterials and eventually move to in vivo studies.

#### 7.1.3 Challenges and Future Outlooks for Antimicrobial Magnetic Hyperthermia Metal Nanomaterials

Nanomaterials with magnetically activated hyperthermic activity are another promising stimuli‐activated technology to combat pathogenic bacteria and fungi. Early research has been encouraging, generating impressive antimicrobial efficacies over short periods. A significant advantage of this technology is the use of magnetic fields, to which human tissue is largely transparent, which is a significant challenge to overcome when translating stimuli‐activated treatments from the lab to a clinically relevant situation. The downsides to magnetic stimulation, however, include decreased accuracy in localization, when compared to laser irradiation, as well as issues with patients who may have certain biomedical devices, such as pacemakers, where exposure to magnetic fields are not a viable solution.

Due to its similar mechanism of action to photothermal nanomaterials, magnetic hyperthermia nanomaterials face similar challenges such as selective targeting of pathogenic micro‐organisms and control of temperature dispersion. Furthermore, the antimicrobial applications of magnetic hyperthermia nanomaterials are only a recent phenomenon, with the first study performed in 2009, as such there are still large gaps in the literature, particularly in vivo studies, to determine any potential cytotoxic side effects or other drawbacks. Additionally, there is also the consideration of heat‐resistant bacteria and fungi. Furthermore, the effect of biological media and potential immobilization of the nanomaterials in vivo are not understood, which could reduce the efficacy or increase aspects of the magnetic exposure, such as field strength and/or frequency, due to losses in heat generation from Brownian relaxation processes. Overall, the initial research into this emerging area indicates specific control of temperature and targeting of pathogenic micro‐organisms is achievable, suggesting this technology has significant potential for growth and development.

Useful future directions for research into magnetic hyperthermia antimicrobial metal nanomaterials include engineering the surface chemistry of the nanomaterials for useful properties such as stability, biocompatibility, and selective targeting of pathogenic micro‐organisms. This should not be a particularly difficult challenge, with a large amount of knowledge already generated in this area largely due to other uses of iron oxide nanomaterials.^[^ ^264^ ^]^ For example, Hayashi et al. demonstrate a simple one‐pot synthesis method for cysteine‐modified Fe_3_O_4_ nanoparticles, to make the nanoparticles hydrophilic and improve biocompatibility.^[^ ^265^ ^]^ Additionally, consideration of the particle design such as size, shape, hierarchical morphology (e.g., core–shell, core–shell–shell, Janus, dumbbell, multicore, yolk–shell, decorated and brush like), composition (hematite/magnetite/maghemite) and potential composites, which can affect the hyperthermia properties of the nanomaterial should be explored in future studies.^[^ ^264^ ^]^ Anisotropic, high aspect ratio magnetic nanomaterials have been shown to have advantages such as improved cell targeting and magnetic properties for other biomedical applications and should also be further explored for antimicrobial applications, which has thus far focused on spherical nanomaterials.^[^ ^266^ ^]^ Alternate shapes such as nanocubes have been shown to possess very high SAR, which is advantageous for in vivo applications where a weaker magnetic field required for activation is beneficial to avoid potential health side effects.^[^ ^267^ ^]^ The primary heat generation mechanism must also be taken into consideration, as Brownian motion of the nanomaterials are environmentally dependent, it is more beneficial to design magnetic hyperthermia nanomaterials, which rely predominately on Neel relaxation for heat generation as they will be more consistent for clinical use where the media surrounding the nanomaterials in vivo will be highly varied due to factors such as location of treatment. Additionally, the materials utilized thus far have been limited mostly to Fe_3_O_4_, while there is evidence in the literature of additional composites of materials which may have beneficial properties, such as CoFe_2_O_4_@Au,^[^ ^268^ ^]^ Cu–Ni,^[^ ^269^ ^]^ Co_1−_ *_x_*Zn*_x_*Fe_2_O_4+_ *_γ_*,^[^ ^270^ ^]^ and Mn*_x_*Zn*_y_*[Fe_2−_ *_z_*Gd*_z_*]O_4_.^[^ ^271^ ^]^

Additionally, a potential avenue of future exploration is through the utilization of the magnetocaloric effect, which is a magnetothermodynamic process, that relies on the disorientation and reorientation of magnetic domains within certain materials.^[^ ^272^, ^273^ ^]^ The effect relies on adiabatic magnetization, where by temperature change occurs within the material without the gain/loss by the surrounding environment. The application of an external magnetic field causes the magnetic dipoles of individual atoms within certain materials to align, reducing the magnetic entropy of the system, which is counteracted by an increase in entropy from the materials lattice, resulting in a rise in temperature due to lattice vibrations.^[^ ^273^ ^]^ Conversely, the removal of the external magnetic field causes magnetic disorientation and an increase in magnetic entropy, resulting in a decrease in temperature; a phenomenon which is primarily utilized for magnetic refrigeration.^[^ ^272^, ^274^ ^]^ Gadolinuium‐based materials, such as Gd_5_Si_2_Ge_2_, are useful due to the Curie temperature of gadolinium occurring near room temperature, where this process works best. Recently, this process has been investigated for biomedical uses including drug delivery^[^ ^273^, ^275^ ^]^ and magnetic hyperthermia treatment of tumors,^[^ ^273^, ^276^ ^]^ where sufficient temperature increases and subsequent destruction of tumors have been reported. The primary advantage of magnetocaloric materials are the large scale of achievable temperature fluctuations, which may allow smaller concentrations of nanomaterials to be used, which reduces potential side effects and is generally considered advantageous. However, following an extensive literature search, we were unable to find applications of magnetocaloric materials for antimicrobial applications, demonstrating a unique gap in the current research of magnetically activated antimicrobial materials.

### 7.2 Magnetophysical Antimicrobial Metal Nanomaterials

The final category of stimuli‐activated antimicrobial nanomaterials are those which are responsive to magnetic fields and utilize a kinetically driven mechanism to rupture individual micro‐organisms or remove communities of micro‐organisms from surfaces. This phenomena is the newest of the stimuli‐activated antimicrobial nanomaterials, hereby referred to as “magnetophysical.” Early studies have primarily focused on the removal of pathogenic biofilms, which are communities of bacteria and/or fungi which form on a surface. Following adherence to the surface, the micro‐organisms excrete extracellular polymeric substances, forming a protective matrix which also enhances adhesion and acts as a protective barrier against factors in the surrounding environment, including antibacterial and antifungal agents.^[^ ^10^, ^12^ ^]^ By rupturing the biofilm matrix, the protective layer is removed and planktonic cells are released into the surrounding medium, which are far easier targets for the majority of antimicrobial agents. While there has been limited research into this area, the studies which investigate nanomaterials for magnetophysical antimicrobial activity have utilized vastly different methods, including gold‐iron microrods,^[^ ^277^ ^]^ Fe_3_O_4_ nanoparticles in combination with additional antimicrobial agents,^[^ ^278^, ^279^, ^280^ ^]^ aggregated Fe_3_O_4_ particles,^[^ ^281^ ^]^ and gallium alloy‐iron liquid metal droplets.^[^ ^282^ ^]^ The mode‐of‐action of these materials varies greatly, as such this review will introduce the concept behind each technology.

#### 7.2.1 Magnetophysical Metal Nanomaterials in Combination with Additional Antimicrobial Agents

**Figure 14:**
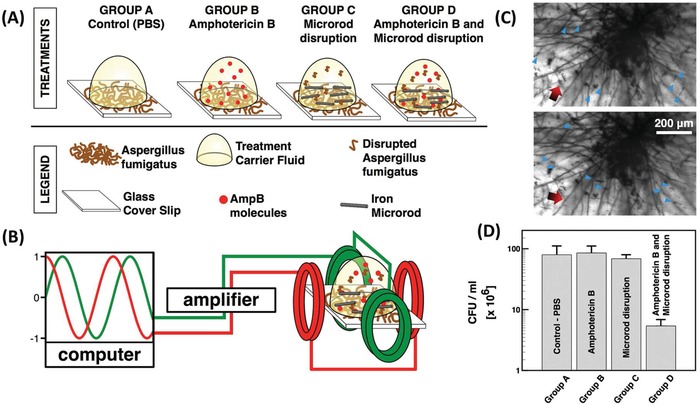
Overview of the experimental setup and magnetophysical activity of Au–Fe–Au microrods against *A. fumigatus* biofilms. A) Schematic depiction of the four treatment groups (from left to right) PBS only (Group A): Amphotericin B (Group B); microrod disruption (Group C); and combined Amphotericin B and microrod disruption (Group D). B) Apparatus used to manipulate the microrods. A computer‐generated signal is amplified before reaching two orthogonally arranged Helmholtz coils. Magnetic fields rotate the particles in the plane of the sample. C) Microrods rotating in and around *A. fumigatus* hyphae. Red arrows indicate the direction of the applied magnetic field. Blue triangles indicate chains of microrods which align parallel with the applied magnetic field. D) Quantification of viable cells through the colony forming unit method. Reproduced with permission.^[^ ^277^ ^]^ Copyright 2017, Elsevier.

A notable early study to utilize magnetophysical properties was conducted by Mair et al. who synthesized Au–Fe–Au microrods as a magnetically activated mechanism to disrupt the biofilm of the pathogenic fungi *Aspergillus fumigatus*.^[^ ^277^ ^]^ The experimental setup involved adding the Au–Fe–Au microrods with and without the addition of the antifungal agent amphotericin B to a healthy *A. fumigatus* biofilm, which were then magnetically actuated through a customized dual Helmholtz coil setup, exposing the particles to a magnetic field of ≈10 mT at a frequency of 10 Hz (**Figure** **14**A,B). The authors reported a substantial decrease in viable cells following treatments with a combination of the microrods and antifungal agent, an improvement on the cell reduction generated from the antifungal on its own (Figure 14). This study demonstrates a simple method of disrupting the biofilm and exposing the planktonic cells, which can be more easily targeted by alternate antimicrobial agents. However, the microrods themselves did not inactivate the cells and the reliance on combinatorial treatment strategies may be a limiting factor for this technology (Figure 14).

**Figure 15:**
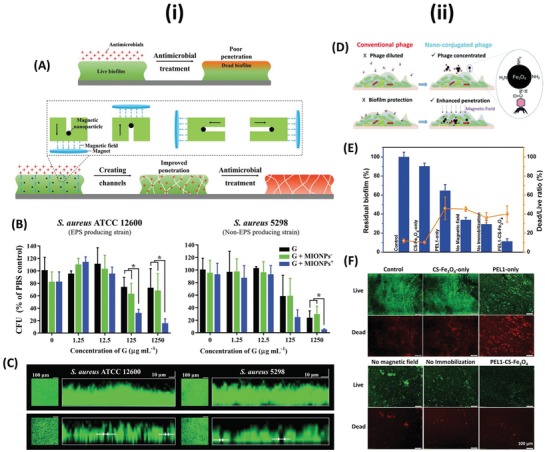
i) The use of magnetic‐iron‐oxide nanoparticles (MIONPs) to create artificial channels within biofilms for enhanced bacterial killing by antibiotics. A) Schematic depiction of the hypothesis that magnetic nanoparticles can be used to engineer artificial channels in infectious biofilms to improve antimicrobial penetration and enhance bacterial killing over the depth of a biofilm. Details not drawn to scale. (Top) Demonstrating the issue that biofilms are poorly penetrable by antimicrobials and often only bacteria at the biofilm surface are killed by antimicrobial treatment, while bacteria residing in deeper layers of a biofilm survive antimicrobial treatment, adding to the recalcitrance of bacterial infections against antibiotic treatment. (Bottom) Inspired by the natural ability of highly motile bacteria to dig channels for the transport of autoinducers, nutrients, and waste products through a biofilm, it is hypothesized that by moving magnetic nanoparticles through a biofilm perpendicular and parallel to a substratum surface, artificial channels can be created that improve antimicrobial penetration and enhance bacterial killing over the depth of a biofilm. B) Numbers of CFUs after exposure to MIONPs (500 µg mL^−1^) without or with 9 min magnetically forced movement in absence (0 µg mL^−1^ gentamicin) or presence of different concentrations of gentamicin for *S. aureus* ATCC 12 600 (left) and *S. aureus* 5298 (right). CFUs are expressed in percentages relative to the number of CFUs after 3 h exposure of biofilms to PBS (0 µg mL^−1^ gentamicin) in a 12‐well plate. C) Overlayer and transverse cross‐sectional CLSM images of 24 h old *S. aureus* ATCC 12 600 and *S. aureus* 5298 biofilms prior to (top) and following (bottom) artificial channel digging by MIONPs (500 µg mL^−1^). Magnetic channel digging (9 min) was initiated after adding 1000 µL of a MIONP suspension (500 µg mL^−1^) to the well, in which a biofilm was grown. Channels perpendicular to the substratum surface appear as black dots on the green‐fluorescent biofilms. Channel widths were measured in cross‐sectional images, as indicated by white arrows. Reproduced with permission.^[^ ^278^ ^]^ Copyright 2019, Wiley‐VCH. ii) Enhanced biofilm penetration for microbial control by polyvalent phages conjugated with magnetic colloidal nanoparticle clusters (CNCs). D) Schematic representation of the principle behind polyvalent phages conjugated with magnetic CNCs, which enables higher localized phage concentrations and enhanced penetration of the biofilm. E) Histograms showing the fraction of the remaining biofilm (area of both live and dead bacteria), and the coverage of control (which was defined as 100%). Assessed by fluorescence microscopy using a live/dead assay. F) Fluorescence microscopic analysis of mixed biofilm disruption. Comparison of the remaining biofilm determined by a live (green)/dead (red) assay without any treatment (control), with free phage treatment only (PEL1‐only) and material treatment only (CS‐Fe_3_O_4_‐only), with both free phage and materials added (no immobilization), and with PEL1–CNC complexes in the presence (PEL1–CS–Fe_3_O_4_) or absence (no magnetic field) of a magnetic field. Reproduced with permission.^[^ ^279^ ^]^ Copyright 2017, Royal Society of Chemistry.

Quan et al. used iron oxide nanoparticles (278 ± 61 nm) as a method to magnetically create artificial channels in the biofilm to allow enhanced dissemination of antimicrobial agents within the biofilm (**Figure** **15**,i).^[^ ^278^ ^]^ Two strains of *S. aureus*, differentiated by their ability to produce EPS, were allowed to grow into a mature biofilm, after which they were treated with iron oxide nanoparticles and varying concentrations of gentamicin. Following treatment, there was an observed four to sixfold reduction in viable cells in the presence of gentamicin (1250 µg mL^−1^) and magnetically activated iron oxide nanoparticles, compared to the gentamicin on its own. Interestingly, they observed no significant difference in cell viability in the presence of the iron oxide nanoparticles when no magnetic field is applied; demonstrating the stimuli‐activatable nature of the treatment which could selectively be “turned on.”

Additionally, Li et al. utilized chitosan‐coated Fe_3_O_4_ colloidal nanoparticle clusters (CS‐Fe_3_O_4_), conjugated with the polyvalent bacteriophage PEL1, to penetrate biofilms of the two Gram‐negative bacterium *P. aeruginosa* and *E. coli* (Figure 15,ii).^[^ ^279^ ^]^ Similar to the previous two studies, the purpose of the CS‐Fe_3_O_4_ nanoclusters were to physically disrupt and penetrate the biofilm, allowing PEL1, the antimicrobial agent in this case, to kill the bacterial cells within. They demonstrated modest killing of the cells within the biofilm (≈40%), however a notable reduction in the overall biofilm coverage (88.7 ± 2.8%).

**Figure 16:**
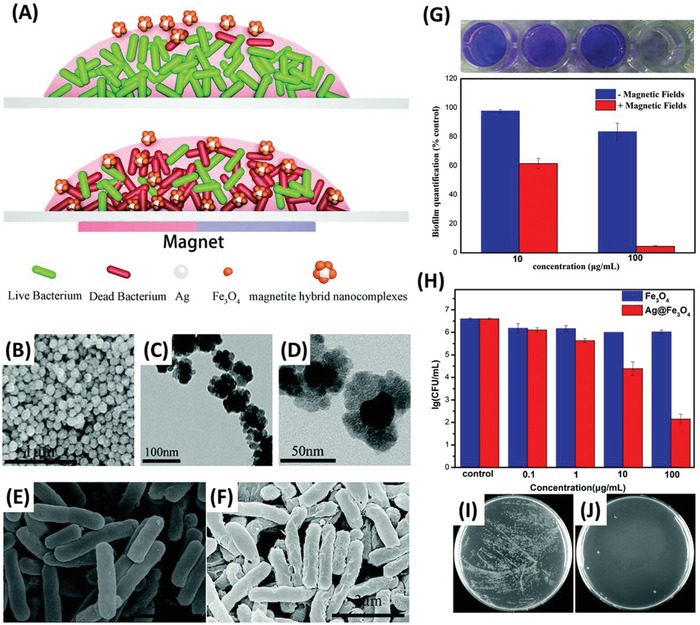
A) The biofilm mode of bacterial growth on a biomaterial surface (top) prevents the penetration of magnetite hybrid nanocomplexes into the biofilm, which can only kill a bacteria on the surface of the biofilm, (bottom) the application of an external magnetic field can facilitate deep penetration of magnetite hybrid nanocomplexes into the biofilm. B) SEM images of magnetite hybrid nanocomplexes. C) TEM and D) HRTEM image of magnetite hybrid nanocomplexes. SEM images of *P. aeruginosa*. E) *P. aeruginosa* in the absence of the magnetite hybrid nanocomplexes, F) *P. aeruginosa* samples after treatment by 100 µg mL^−1^ magnetite hybrid flowers for 30 min. G) Antibacterial properties of magnetite hybrid nanocomplexes against *P. aeruginosa* biofilms with or without a magnetic field. (Top) Crystal violet stained biofilms, (bottom) the percentage survival of *P. aeruginosa* biofilms after being treated with different concentration of magnetite hybrid nanocomplexes with or without a magnetic field (determined via absorbance values at 590 nm). H) Bacterial viability of *P. aeruginosa* treated by iron oxide or magnetite hybrid nanocomplexes at different concentrations. Representative images of plate counts of bacterial colonies formed by *P. aeruginosa* I) in the absence of magnetite hybrid nanocomplexes and J) after being treated with 100 µg mL^−1^ magnetite hybrid nanocomplexes for 30 min. Reproduced with permission.^[^ ^280^ ^]^ Copyright 2019, Royal Society of Chemistry.

Furthermore, Zhang et al. synthesized nanocomposites, ≈50–70 nm in diameter, composed of an inner core of nanosilver surrounded by an outer shell of Fe_3_O_4_.^[^ ^280^ ^]^ In this case, the Fe_3_O_4_ enabled the nanocomposites to be magenetically activated for biofilm penetration, while the nanosilver acted as the antimicrobial agent. Testing against biofilms composed of *E. coli* and *P. aeruginosa* bacteria, they demonstrated significant reduction of the biofilm through crystal violet staining and viable cells, through CFU determination via plate counting (**Figure** **16**).

#### 7.2.2 Magnetophysical Antimicrobial Metal Nanomaterials without Additional Antimicrobial Agents

**Figure 17:**
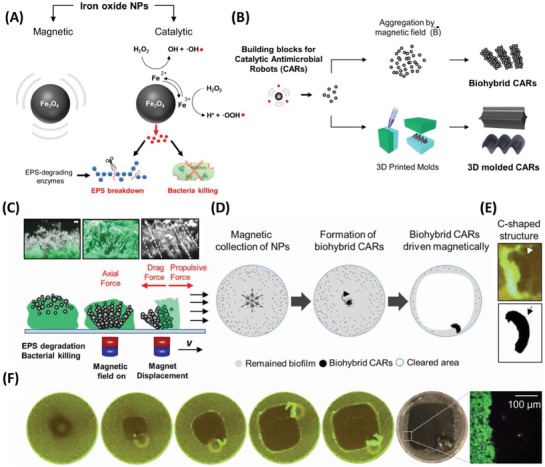
Catalytic and magnetic iron oxide nanoparticles (NPs) as building blocks for small‐scale robots designed for biofilm killing and removal. A) Diagram depicting the magnetic‐catalytic NPs and their bacterial killing and EPS degradation mechanisms via reactive free radicals generated from hydrogen peroxide (H_2_O_2_) via peroxidase‐like activity. The EPS degrading activity was enhanced by addition of mutanase/dextranase to digest extracellular glucans. B) Catalytic‐magnetic NPs in suspension served as multifunctional building blocks to form catalytic antimicrobial robots (CARs). In the first CAR platform, biohybrid CARs with bristle‐like structures were assembled from NPs suspended in H_2_O_2_ and mutanase/dextranase solution by a permanent magnet attached to a micromanipulator and used to remove biofilms from accessible surfaces. In a second platform, catalytic‐magnetic NPs were embedded into gels to form 3D molded CARs having specialized vane and helicoid structures. C) Experimental and representative schematic depiction of the orthogonal view of biofilms treated with biohybrid CARs, which demonstrate the formation of a rod‐like superstructure forming bristles following magnetic actuation and the resulting physical removal of the biofilm through magnetic displacement. Scale bar indicates 10 µm. D) Diagram depicting the cleaning of biofilm‐coated surfaces by magnetically controlling the sweeping of the biohybrid CARs. E) The biohybrid CAR morphed into C‐shaped aggregate (see arrows) as it moved over the surface to plow through and remove biofilm (labeled with SYTO 9 green fluorescence). F) Fluorescent images indicating complete cleaning of an *S. mutans* biofilm (labeled in green) grown on a glass surface by using biohybrid CARs sweeping effect. Reproduced with permission.^[^ ^281^ ^]^ Copyright 2019, AAAS.

New research has presented magnetophysical nanomaterials that can both physically destroy the protective biofilm and kill the individual cells within, without the need for additional antimicrobial agents. For example, Hwang et al. utilized Fe_3_O_4_ nanoparticles, which following magnetic actuation, aggregated to form larger assemblies.^[^ ^281^ ^]^ They termed these macrostructures catalytic antimicrobial robots (CARs), which were magnetically responsive, able to physically disrupt and remove the biofilm, while the catalytic properties of the particles kill the individual cells (**Figure** **17**). Through the application of a magnetic field, they demonstrated a physical removal of a bacterial biofilm formed by the Gram‐positive bacterium *Streptococcus mutans*, showing a significant decrease in biomass and viable cells. Through control over the applied magnetic field, they were able to demonstrate the control over the directionality of the treatment in two dimensions (Figure 17F). The applications of these particles in 3D space was also explored, utilizing 3D printed molds to form microscale vane‐like and helicoid shaped magnetically activated CARs, which were driven by Helmholtz coils to physically scrub off a biofilm blockage of a tube. Furthermore, the utility of this technology was shown to successfully remove biofilms on teeth, a relevant application, over a time period of seconds.

**Figure 18:**
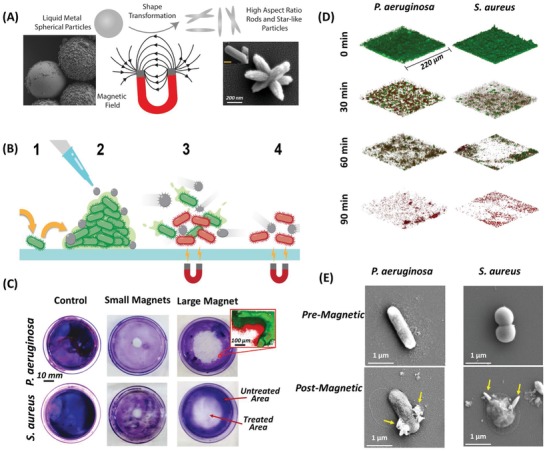
Magnetic actuation shape‐transformation of liquid metal particles and subsequent antibiofilm activity. A) Scanning electron micrographs and pictorial representation of the magnetically induced shape transformation of galinstan‐iron particles from spherical (left) to high aspect ratio rods and star‐like shapes (right) (unpublished). B) Schematic diagram representing the magnetophysical antibiofilm activity of galinstan‐iron liquid metal droplets. (1) Bacteria form biofilm on surface. (2) Magnetic liquid metal particles are added to the pathogenic biofilm. (3) Particles shape‐transform following magnetic exposure. 4) High aspect ratio nanoparticles remove the pathogenic biofilm and rupture individual bacterial membranes. C) Illustrating the spatial control of antibiofilm activity of magnetophysical particles, control (left) and treated biofilms with a small (middle) and large (right) magnet, following staining with crystal violet. The zoomed inset shows a CLSM image of the periphery of the treated area. D) CLSM images of *P. aeruginosa* (left) and *S. aureus* (right) biofilms following 30 min increments of magnetic field exposure. E) High‐magnification SEM images of liquid metal particle‐cell interactions. *P. aeruginosa* (left) and *S. aureus* (right) cells before (top) and after (bottom) exposure to a magnetic field. Yellow arrows indicate particle–cell interactions and significant damage to the cell membrane. Reproduced with permission.^[^ ^282^ ^]^ Copyright 2020, ACS Publications.

Uniquely different again, Elbourne et al. utilized magneto‐responsive gallium‐based liquid metal droplets.^[^ ^282^ ^]^ The authors combined nanoscale iron particles with the eutectic gallium–indium–tin alloy, commonly known as “Galinstan,” which was liquid at room temperature, to allow the material to be magneto‐responsive. When exposed to a low‐intensity rotating magnetic field, the magnetic Galinstan droplets undergo a shape‐transformation from spheres to high aspect ratio rods and star‐like particles with nanoscale sharp edges (**Figure** **18**). These particles were applied to biofilms formed by the Gram‐positive *S. aureus* and Gram‐negative *P. aeruginosa* bacteria, following magnetic actuation, the biomass and percentage of viable cells were significantly reduced (Figure 18). The removal of the biofilm was caused by the movement of the particles under the force generated by the magnetic inclusion, effectively removing the majority of the adherent biomass (Figure 18). The movement of the particles exerted physical force onto the bacterial cells, which due to the nanoscale protrusions of the particles, appeared to pierce the bacterial cell wall, breaking the cell membrane and inactivating the pathogenic bacteria (Figure 18). This process differs from the earlier cases, which rely on a chemical mechanism for inactivation of the individual cells. Furthermore, the capacity to spatially control the treatment area, using an applied magnetic field was demonstrated. This can be visualized in Figure 18C, which demonstrates the control over the size of the antibiofilm activity in response to the size of the magnetic field.

#### 7.2.3 Challenges and the Future Outlook for Antimicrobial Magnetophysical Nanomaterials

Magnetophysical micro‐ and nanomaterials present as the newest stimuli‐activated materials, which demonstrate antimicrobial properties, with a primary focus on antibiofilm activity. These materials can be actuated through the application of an external magnetic field, however there are a range of different designs with differing biofilm removal and antimicrobial efficacy. This technology demonstrates an obvious application in combination with additional antimicrobial agents, such as antibiotics, antifungals, and bacteriophage, among others, to physically disrupt the biofilm and allow the targeting of more susceptible planktonic cells. Furthermore, aggregations of magnetic particles can be used to scrub biofilms from 2D and 3D surfaces via a physical mechanism. Finally, magnetic liquid metal materials have been shown to physically remove biofilms as well as inducing significant cell inactivation through magnetically actuated shape‐transformed, nanoscale high aspect ratio particles. The advantage of this class of stimuli‐activated nanomaterials is the physical mode of action, which proves effective in removing biofilm communities which are typically resistant through a protective matrix primarily composed of EPS, cells and additional components. This physical antimicrobial mechanism is less susceptible to the development of resistance by pathogenic micro‐organisms, compared to traditional chemical‐based approaches. A major advantage of magnetophysical particles is the capacity to spatially control the treatment area, through control over the applied magnetic field. Currently, this level of spatial control has only been observed in two dimensions, however, with continual advances in noncontact control of magnetic particles in 3D space,^[^ ^283^ ^]^ there is potential that the spatial control demonstrated in two dimensions can be translated to 3D space, ensuring such particles are clinically relevant. Additionally, antimicrobial activity appears to be controlled by the absence/presence of an external magnetic field, demonstrating the temporal control of the treatment. However, as the newest of the stimuli‐activated technologies, magnetophysical materials need to address a number of challenges such as control in 3D space, systematic cytotoxicity studies, activity against planktonic cells, pathogen selectivity, and comprehensive in vivo studies. Overall, this technology has considerable scope for exploration and future research should be supported to determine its capabilities. A useful future avenue could be combining this technology with additional antimicrobial technologies, such as other stimuli‐activated antimicrobial agents, for synergistic approaches to removing biofilms and inactivating individual pathogenic micro‐organisms.

## 8 Conclusions and Future Outlooks

In the wake of the rapid rise of AMR pathogenic bacteria and fungi, metal nanomaterials offer a range of unique solutions to combat them. In conjunction with recent and anticipated improvements in nanotechnology, such as increased control, scalability and reduced cost of synthesis techniques, it is reasonable to expect current knowledge and prospects of antimicrobial, metal nanomaterials to improve exponentially in the future. Importantly, the discovery and subsequent development of stimuli‐activated antimicrobial nanomaterials, which can be turned “on” or “off” on demand, are an exciting avenue to replace or improve upon current treatment methods, or to be used in combination.

Photocatalytic nanomaterials are the most comprehensively studied stimuli‐activated treatment, against both bacteria and fungi, which demonstrate impressive antimicrobial activity. However, this technology faces challenges to reduce the length of the treatment time and modify relevant parameters, such as the size and composition of the metal nanomaterial to shift the required excitation wavelength into the NIR region, which is necessary for clinical applications, while maintaining sufficient redox potential to drive the generation of antimicrobial ROS.

Comparatively, metal nanomaterials that induce localized hyperthermia, stimulated by exposure to either light or magnetic fields, show significant promise even for deep infections, as they can be activated by wavelengths within the NIR range, through the biological windows, or magnetic fields, to which human tissue is largely transparent. Furthermore, successful targeting and inactivation of specific pathogenic bacterial and fungal species has been demonstrated, in clinically relevant exposure times. However, these technologies are still very much prospective and future research requires systematic studies and in vivo trials to further optimize and assess the “real world” applications of such treatments. Additionally, the interactions between stimuli‐activated thermal induced nanomaterials and fungi have been notably understudied and is an obvious gap in the current research.

Interestingly, magnetophysical metal nanomaterials have been shown as the latest innovation in stimuli‐activated antimicrobial nanomaterials. Our recent work, among others in the field, has shown that this class of nanomaterials can remove thick pathogenic biofilms with great efficacy, through a magnetically actuated, kinetically driven and nonselective mechanism.^[^ ^282^ ^]^ The primary advantage of this class of nanomaterial is the disruption of the biofilm associated pathogenic micro‐organisms, which are notoriously difficult to remove and treat. While this technology is only in its infancy and there is a great deal of research required for improvement, there is exceptional success in using multidimensional approaches as discussed in Koo et al.^[^ ^284^ ^]^ Current magnetophysical technologies still requires further control in 3D space, systematic cytotoxicity studies, assessment of antimicrobial efficacy against planktonic cells, pathogen selectivity, and comprehensive in vivo studies.

Overall, stimuli‐activated, metal nanomaterials with antimicrobial properties have the potential to be a unique replacement to conventional antimicrobial strategies, which are beginning to fail. With focused efforts into research, it is reasonable to predict that one of, or a combination of, the technologies evaluated in this review have the potential to become a viable treatment method in clinical settings in the future. Still, large hurdles need to be overcome specific to each technology, as outlined above. Broadly, future research in this area will benefit from exercising well‐thought‐out design practices to synthesize metal nanomaterials, which address particular gaps in the literature, as well as investigations into the specific mechanisms which cause cell death. There are some inconsistencies within the reported literature, with some antimicrobial efficacy reported as a percentage; in contrast some authors report a log reduction. Additionally, the concentrations of nanomaterials used for antimicrobial testing are highly variable and can be tested against pathogenic micro‐organisms for different time scales using disparate parameters of light and magnetic stimuli, such as wattage and frequency. General considerations will be required for stimuli‐activated, antimicrobial nanomaterials to be used in clinical applications. To this end, the following key criteria should be comprehensively addressed: high antimicrobial efficacy, pathogenic micro‐organism selectivity, low cytotoxicity, localizable to target infected areas, spatial and temporal control, and an uncomplicated and relevant method of administration in clinical settings. By addressing these criteria and through continued innovation, metal nanomaterial based therapeutic strategies have great potential as the next generation of antimicrobial technologies for the treatment of planktonic and biofilm associated pathogenic micro‐organisms.

## Conflict of Interest

The authors declare no conflict of interest.
